# gender.neutral@work.de: An experimental approach to the discrimination of nonbinary individuals during job applications

**DOI:** 10.1111/bjso.70081

**Published:** 2026-04-28

**Authors:** Lou Dörr, Elena Ball, Pia Vogel, Claudia Niedlich, Melanie C. Steffens

**Affiliations:** ^1^ Social, Environmental, and Economic Psychology, Faculty of Psychology RPTU University Kaiserslautern‐Landau Landau Germany

**Keywords:** employment discrimination, experimental design, gender discrimination, nonbinary, stereotyped attitudes, transgender, workplace diversity

## Abstract

For many nonbinary individuals, disclosing their pronouns and preferred forms of address when applying for a job is necessary to avoid being misgendered. The request to be referred to in a gender‐neutral way may trigger stereotypes and result in discrimination. Simulating recruitment scenarios, we test the effects of an applicant's request for gender‐neutral address and avoidance of pronouns compared to binary‐gendered alternatives. We hypothesize that applicants with a gender‐neutral request would be discriminated against compared to applicants requesting binary‐gendered pronouns. In a pre‐registered pre‐experiment with a convenience sample (*N* = 248), we found that applicants with a gender‐neutral request were misgendered more often than applicants requesting binary‐gendered pronouns and forms of address. No other indicators of discrimination were found, possibly due to the convenience sample. The reviewed experiment tested the hypotheses in a more diverse sample (*N* = 1275), adding openness towards nonbinary gender (ONBG) as a moderator variable and investigating spontaneous stereotype content. The findings demonstrated that applicants with a gender‐neutral request were discriminated against compared to masculine‐request applicants during the initial written application stage, with bias being moderated by ONBG. We discuss implications for understanding and reducing discrimination against nonbinary applicants in the work context.

## INTRODUCTION

Whenever people meet someone new, their gender identity is typically inferred (correctly or incorrectly) guided by indicators such as appearance, name or explicit statements. Based on the gender/sex binary (Morgenroth & Ryan, 2021), these guesses often fall into two (cis‐) normative categories: women and men. As a result, to prevent being misgendered, most nonbinary people need to disclose preferred forms of address, which may expose them to stereotyping and discrimination. Social–psychological research has traditionally adhered to a binary understanding of gender/sex, overlooking the experiences of nonbinary individuals (Morgenroth & Ryan, 2018). The present research sought to compare stereotyping and discrimination of people stating their preferred forms of address and pronoun use, thus indicating a nonbinary or binary gender identity. As a critical situation for examining potential discrimination in first encounters, this research is embedded within the context of job applications.

We first review the reported experiences of discrimination made by trans and nonbinary people in the workplace, differentiating formal and interpersonal forms. Expanding beyond negative attitudes as causes of discrimination, we consider congruity models of gender‐based discrimination.

### Experiences of nonbinary individuals

Being nonbinary describes the experience of individuals who do not (wholly, solely or consistently) align with a male or female identity. This encompasses those who fall beyond a binary conceptualization of gender and those who do not identify with any gender (Galupo et al., 2017). As these identities often do not match people's gender assigned at birth, nonbinary individuals are oftentimes included under the broader trans*^1^ umbrella. Despite their differences, the distinction between nonbinary people and binary trans individuals (i.e. trans women and men) is hardly made in research, precluding a differentiated understanding (Burke et al. 2026; Worthen, 2021).

In the work context, rates of discrimination (European Union Agency for Fundamental Rights, 2020a, 2020b, 2020c, 2020d; Frohn & Heiligers, 2024; Fütty et al., 2020) and unemployment (Fütty et al., 2020) faced by trans* individuals are reportedly high, negatively impacting their well‐being (Cancela et al., 2024; Schmitt et al., 2014; Tebbe et al., 2019) and work (Dhanani et al., 2018). By not conforming to the norm of binary gender (i.e. being men or women), nonbinary people are likely to be treated differently than binary‐gendered individuals (Davidson, 2016).

Binary‐gendered information is ubiquitous and used habitually, even in professional contexts, from language use like formal address and pronouns to gendered spaces and dress codes. Consequently, individuals outside of this binary frequently experience misgendering (Dowers et al., 2019; Matsuno et al., 2024; Truszczynski et al., 2022), which refers to the misclassification of a person's gender and ‘can include intentional or unintentional acts of using the wrong name, pronoun, or gendered language to refer to someone’ (Jacobsen et al., 2024, p. 817). Contrasting it to formal discrimination (e.g. hiring and promotion), which is oftentimes illegal, we consider misgendering a form of interpersonal discrimination. This comprises more subtle forms of discrimination in social interactions that surface in nonverbal, paraverbal and verbal behaviour, such as shorter interaction length and avoiding eye contact during a job interview (Hebl et al., 2002) or reduced willingness to engage in work‐related contact (Niedlich et al., 2022; Steffens, Niedlich, et al., 2019). Interaction time, word count and the affect displayed during interactions have been identified as indicators of interpersonal discrimination in face‐to‐face interactions (Hebl et al., 2002; King & Ahmad, 2010; King et al., 2006; Singletary & Hebl, 2009), including job application interviews.

Whereas discriminatory behaviour targeting nonbinary individuals thus is expected to be frequent, not all individuals should be equally likely to discriminate against them. Individual differences in the motivation to act without prejudice (Devine et al., 2002) could be a proxy for distinguishing people who differ in their willingness to (not) discriminate against nonbinary individuals. However, the most adequate moderator should be captured by a scale explicitly developed for a range of nonbinary experiences: the openness towards nonbinary gender (ONBG) scale (Molin et al., 2021). To date, it was mostly used in gender categorization research (e.g. Gallagher et al., 2025; Weißflog & Grigoryan, 2024), but non‐acceptance of nonbinary identities could also encourage individuals to not respect gender‐neutral language or reject individuals outside the gender binary. In a pre‐study for this paper, the ONBG was the strongest predictor for the support of equality policies and political solidarity with nonbinary people (see Table S1).

Beyond habits and individual biases, contextual factors also play a role in discrimination. Research differentiating sex‐typed job contexts supports congruity models of gender discrimination, such as the lack‐of‐fit model (Heilman, 1983, see also Eagly & Karau, 2002): Hireability judgements are influenced by the perceived mismatch between the attributes required for a job and the characteristics ascribed to an individual, which are influenced by (binary) gender stereotypes (Di Stasio & Larsen, 2020; Koch et al., 2015; Niedlich et al., 2015; Sczesny et al., 2022). Evidence of the preferential treatment of male applicants for male‐typed jobs provides strong support for congruity models. In contrast, findings on female‐typed and non‐sex‐typed jobs are less consistent (for meta‐analyses, see Koch et al., 2015; Paustian‐Underdahl et al., 2014; see also Manzi, 2019). Consequently, we will focus on male‐typed jobs to test whether the lack‐of‐fit model applies to nonbinary applicants. To this aim, it is necessary to know their perceived attributes.

### Stereotypes about nonbinary individuals

The stereotype content model (SCM, Fiske et al., 2002) postulates how stereotypes shape person perception. It describes the content of stereotypes along two fundamental dimensions (the Big Two): warmth and competence (parallel to communion and agency, Fiske, 2018). In the context of gender, stereotyping generally follows an asymmetrical and complementary pattern: traditionally feminine characteristics are linked to high warmth but low competence, whereas traditionally masculine characteristics are perceived as indicating low warmth but high competence (for the relation between stereotypes and social roles, see Koenig & Eagly, 2014).

Nonbinary individuals present a theoretically relevant social group for testing these assumptions, as their ascribed attributes could align with one of several patterns: they could be perceived as being (a) highly androgynous (i.e. high in both warmth and competence), and thus potentially fitting sex‐typed jobs; (b) moderately androgynous, positioned between women and men (i.e. higher in competence but lower in warmth than women, higher in warmth but lower in competence than men), similar to lesbian women and gay men (Asbrock, 2010; Brambilla et al., 2011; Klysing et al., 2021); or (c) genderless (i.e. low on both warmth and competence) and thus apparently unfit for stereotypically gendered jobs.

Looking at the general determining factors of warmth and competence in the SCM (competition for warmth and status for competence), it seems unlikely that nonbinary individuals would receive high ratings on both dimensions. Their perceived low societal status (e.g. Jacques et al., 2022) should result in lower competence ratings. Additionally, nonbinary people might be perceived as a threat to the traditional gender/sex binary (Morgenroth & Ryan, 2021) and thus high in competition (see Kervyn et al., 2015), which could result in lower warmth ratings. Empirical findings on the perception of nonbinary people are mixed: They were stereotyped as lower in warmth and competence than women (Hansen & Żółtak, 2022; Jacques et al., 2022), lower (Hansen & Żółtak, 2022) or similar (Jacques et al., 2022) in both to men, or similar to both women and men (Weißflog & Grigoryan, 2024). Interestingly, compared with trans women, nonbinary individuals were rated as less feminine and more masculine, but lower in competence, sociability and morality (i.e. warmth) (Taylor & Fasoli, 2022). This suggests that the link between femininity/masculinity and the Big Two may be weaker or different for nonbinary individuals.

One possible constraint of the stereotype content model is that it may not (fully) capture the stereotypes that people apply to groups (A. Koch et al., 2016; Nicolas et al., 2022). Recent findings have highlighted unique stereotypes applied to trans women and men, for example, (mentally) ill (also nonbinary individuals, McCarty & Burt, 2024), (ab)normal, deviant, confused, gay, disgusting (Gazzola & Morrison, 2014; Howansky et al., 2021; Reed et al., 2015; Van Borm & Baert, 2018). We therefore aim to investigate spontaneously applied stereotypes through open‐ended responses, as proposed in the spontaneous stereotype content model (SSCM, Nicolas et al., 2022). In addition to assessing unique stereotypes not represented in traditional scales, this approach allows insights into the representativeness of established stereotype dimensions, indicating the strength of a perceiver's spontaneous association between them and a target group (Nicolas et al., 2022).

Recent (field) experiments manipulating pronouns (see Eames, 2024; Landry & Yacknovets, 2022; McGonagill, 2023; Tomlin, 2024) and using email correspondence (e.g. Milkman et al., 2015; Tomlin, 2024) emphasize the importance of early decision‐making situations within and beyond the context of job applications. Building on them, our research question is how nonbinary job applicants are perceived and potentially discriminated against, compared to binary‐gendered applicants. More specifically, we aim to investigate formal and interpersonal discrimination against people requesting to be referred to in a gender‐neutral way in the first stage of the job application process (see Milkman et al., 2015) and explore stereotypes about these applicants. Concurrently, boundaries of established explanations for gender discrimination in the job context are examined. Aiming to unpack the components of discrimination against nonbinary applicants (see Figure S1), we focus on raters' perspective, starting with minimal, well‐controlled information: written requests asking to be referred to in either a gender‐neutral or binary‐gendered way.

### The present research

Across two experiments simulating a recruitment scenario with written information only, we test the hypotheses that nonbinary individuals face hiring discrimination as well as interpersonal discrimination, including being misgendered. A pre‐registered pre‐experiment using a convenience sample initially tested these (plus additional) hypotheses. We used email applications that contained forms of address and pronouns preferred by the applicant to indicate a binary versus nonbinary gender identity. Participants were told they were responsible for hiring decisions and email correspondence both with the applicant and a colleague. The registered experiment aims to replicate the pre‐experiment with a more diverse sample, include a potential moderator (openness to nonbinary gender) and additionally measure spontaneous stereotypes.

## PRE‐EXPERIMENT

The aim of the pre‐experiment was to generally test our procedure and measures, empirically examining the stereotyped perception of nonbinary people (on warmth and competence) plus formal and interpersonal discrimination. While discrimination in recruitment could be based on negative attitudes, a perceived difference between stereotypes and required attributes of a male‐typed job could also lead to rejection of an applicant (i.e. lack‐of‐fit). Because exclusion (66.8%) and withdrawal of customer contact (12.3%) are especially pronounced for trans* employees in Germany (Frohn & Heiligers, 2024), we chose IT consultant as a male‐typed job (Misersky et al., 2014), where this kind of discrimination could occur.

In addition to our hypotheses and measures, the pre‐experiment included additional ones not of interest here. Table S2 juxtaposes the pre‐registration and the present report.

We expected formal discrimination of nonbinary applicants (H1): Applicants requesting to be referred to in a gender‐neutral way will be hired less often for the male‐typed job than applicants requesting to be referred to in a masculine way. We also expected interpersonal discrimination towards nonbinary applicants: Applicants requesting to be referred to in a gender‐neutral way will be misgendered more often (H2a) and met with a lower willingness to engage in work‐related contact (H2b). Extending existing findings on face‐to‐face discrimination to emails, we also expected them to be treated less politely (i.e. response emails being shorter, H2c, and showing less positive affect, H2d) than applicants with a binary‐gendered request. In addition, we expected moderately androgynous or genderless stereotyping of nonbinary individuals (H3): Applicants who request to be referred to in a gender‐neutral way are rated lower in competence than in those with a masculine request and lower in warmth than in those with a feminine request.

### Method

#### Participants, manipulation and design

The pre‐study was conducted with a German‐speaking convenience sample of lay people. Participants were recruited through university email lists and social media platforms. As an incentive, participants could take part in a raffle or receive course credit. Excluding four participants who did not consent to data usage or who were under the age of 18, the final sample consisted of 248 German‐speaking individuals (164 women, 73 men, 1 neither, 4 not categorizing, 3 using their own label, 3 choosing not to respond), aged 19–70 years (*M* = 30.32, *SD* = 10.83), 48% of whom indicated to know a nonbinary person. About half were students, and average political orientation (scale: *1 – left to 11 – right*) was below the scale midpoint, *M* = 3.83 (*SD* = 1.97), with 79.4% left‐leaning (for additional demographics, see Online Supplement, OS, Table S4). As pre‐registered, additional robustness checks excluded participants who did not infer the applicant's intended gender identity (total *n* = 45), failed the attention check (*n* = 4) or took too long (3 *SD* above mean time taken, *n* = 1). The pattern of non−/support of the hypotheses remained identical (for details, see OS).

In a between‐subjects design, participants were randomly assigned to one of four conditions. Applicant gender was subtly indicated, first, by using several (Wells & Windschitl, 1999) comparable names: gender‐neutral: Alex/Charly/Lou; feminine: Alexandra/Carla/Louisa; masculine: Alexander/Carl/Louis (e.g., Rudolph et al., 2007). Second, we included linguistic cues, namely pronouns and asking for corresponding forms of address, as shown in Table 1. Since there is no clearly established gender‐neutral or gender‐expansive pronoun for people in German, nonbinary people often ask not to be referred to with third‐person pronouns (see OS for exact wording). Two conditions thus used gender‐neutral names, but differed in that one did not include a request to be addressed in a certain way, serving to detect the influence of said request in general (see Tomlin, 2024). Because our analyses have more power if we compare only the gender‐neutral condition with the feminine and masculine condition, we used a one‐factorial three‐level design for the main analyses (see OS for analyses including the no‐request condition).

**TABLE 1:** Experimental conditions in the pre‐experiment.

Indicators	Request			
	Gender‐neutral	Feminine	Masculine	[No request]
Names	Alex/Charly/Lou	Alexandra/Carla/Louisa	Alexander/Carl/Louis	Alex/Charly/Lou
Pronouns	No pronouns	She/her	He/him	–
Forms of address	Alex/Charly/Lou Müller	Ms. Müller	Mr. Müller	–

Sensitivity power analyses (see pre‐registration for a priori power analyses) indicate that medium‐size effects of *f* = .23 could be detected in the one‐way three‐level ANOVAs with a power of 1−β = .90 and α = .05.

#### Procedure

After consenting, participants examined a short job posting for the male‐typed job of IT consultant (see OS for details), including customer contact. Participants then read the job application email of one applicant and were asked to make a dichotomous hiring decision. Subsequently, to examine which form of address and pronouns were used, participants communicated their decision to the applicant (testing form of address) and to their substitute (testing use of pronouns and gendered language). Addressing a third party in a separate email also reduces the likelihood of socially desirable behaviour. Throughout the process of composing these emails, participants could see the application email cited at the bottom, thus being reminded of the gendered/non‐gendered request of address. After that, participants judged the applicant on several scales, and covariates were collected before debriefing.

#### Measures

##### Formal discrimination

Formal discrimination was measured as the decision to hire the applicant (yes/no).

##### Interpersonal discrimination

Interpersonal discrimination was assessed by manually coding emails as correctly gendered (0), incorrectly gendered at least once (1) or missing. We further coded whether the applicant was addressed or referred to in a gender‐neutral (1), feminine (2) or masculine (3) way. As a more subtle direct behavioural measure, we assessed (non‐)politeness through *interaction length* (word count) and level of *positive affect* displayed in the emails, using the text analysis software Linguistic Inquiry and Word Count (DE‐LIWC2015, Meier et al., 2018; Pennebaker et al., 2015). Furthermore, we assessed the willingness to engage in work‐related contact with three items following Steffens, Niedlich, et al. (2019, e.g. ‘I can well imagine conducting a consultation meeting for a customer together with the applicant’, Cronbach's α = .84). If not mentioned otherwise, all scales were endpoint‐labelled (1—*do not agree at all* to 7—*fully agree*) and averaged.

##### Stereotypes

Competence and warmth were assessed using two items each from Abele et al. (2016), scale 1–5, for example, *not at all friendly* to *very friendly* (warmth: friendly, trustworthy: *r* = .46; competence: competent, self‐confident: *r* = .54). Using a comparable scale, ratings of *masculinity* and *femininity* were also collected (Martin & Mason, 2022; Steffens et al., 2017; Steffens, Niedlich, et al., 2019).

##### Covariates

Beyond *political orientation*, we measured individual differences in the *motivation to act without prejudice* with 10 items from Banse and Gawronski (2003), for example, ‘If I have a prejudiced thought or feeling, I keep it to myself’, Cronbach's α = .74, scale 1–5.

##### Supplementary variables

Other possible mechanisms of discrimination like *processing fluency* (Lick & Johnson, 2015), *dehumanization* (see Martin & Mason, 2022) and covariates (e.g. *gender essentialism*) were explored. Given that none of the potentially explanatory variables had the expected effect, we report them in the OS (Tables S5–6).

##### Quality checks

Participants were asked how familiar they were with the term nonbinary gender (scale 1—*not familiar* to 5—*very familiar*), and whether they knew any nonbinary people. Manipulation checks included questions about the applicant's gender/sex, gender identity, transness and non‐usage of pronouns due to political reasons. An attention check was included.

### Results

An overview of descriptive statistics and statistical tests is presented in Tables 2 and 3. In short, applicants requesting to be referred to in a gender‐neutral manner were misgendered more often, but no other evidence of discrimination was found. In detail, while most participants indicated they would hire the applicant, we found no statistically significant relationship between applicant request and hiring. Thus, Hypothesis 1 was not supported.

**TABLE 2:** Overview of descriptives and statistical tests of the pre‐experiment for binary outcomes (hiring decisions and misgendering).

Measure	Condition				Statistical test (3 conditions)	*p*
	Gender‐neutral (*n* = 67)	Feminine (*n* = 56)	Masculine (*n* = 65)	[No request] (*n* = 60)		
Formal discrimination						
1. Hiring decision	80.60% (54)	67.86% (38)	72.31% (47)	85.00% (51)	χ^2^(2, *N* = 188) = 2.71	.259
Interpersonal discrimination						
2. Misgendering						
Response email	15.15% (10) *n* = 66	5.45% (3) *n* = 55	3.13% (2) *n* = 64	GN: 23.73% (14) F: 20.34% (12) M: 55.93% (33) *n* = 59	Fisher's exact test	< .001
					GN – F GN – M	.004 .001
Email to colleague	24.62% (16) *n* = 65	5.56% (3) *n* = 54	1.56% (1) *n* = 64	GN: 25.86% (15) F: 15.52% (9) M: 58.62% (34) *n* = 58	Fisher's Exact Test	< .001
					GN – F GN – M	.001 .001

*Note*: Statistical tests for the three conditions with a request for specific use of pronouns/formal address (gender‐neutral, feminine, masculine). For misgendering, *n*_*s*_ were specified due to missings.

Abbreviations: F, feminine; GN, gender‐neutral; M, masculine.

**TABLE 3:** Means, standard deviations, and analyses of variance of the pre‐experiment for interpersonal discrimination and stereotypes.

Measure	Condition								*F*(2, 185)	*p*	η^2^
	Gender‐neutral		Feminine		Masculine		[No request]				
	*M*	*SD*	*M*	*SD*	*M*	*SD*	*M*	*SD*			
Interpersonal discrimination											
1. Willingness to work together	5.59	1.33	5.13	1.34	5.06	1.39	5.21	1.24	2.95	.055	.03
2. Politeness											
Word count	41.97	16.26	42.46	21.32	44.00	19.49	42.18	18.17	0.20	.817	<.01
Positive affect	13.51	4.74	14.06	4.63	12.10	4.93	12.42	4.21	2.78	.065	.03
Stereotypes											
3. Competence	4.16	0.77	3.95	0.74	4.00	0.84	4.11	0.66	1.21	.300	.01
4. Warmth	4.11	0.74	4.03	0.64	3.95	0.67	4.06	0.58	0.88	.415	.01
5. Femininity	2.90	0.68	3.64	0.62	2.20	0.85	2.77	0.72	59.43	<.001	.39
6. Masculinity	2.91	0.54	2.32	0.66	3.67	0.79	3.17	0.67	61.86	<.001	.40

*Note*: Statistical tests for the three conditions with a request for specific use of pronouns/formal address (gender‐neutral, feminine, masculine).

*Misgendering* of applicants with a gender‐neutral request happened in 15% of emails to the applicant and in 25% of emails to the colleague. In contrast, binary‐gendered applicants were gendered correctly (>94%, all instances of misgendering being gender‐neutral forms). Two‐tailed Fisher's exact test (because some cell frequencies were <5) for the response email was significant, *p* < .001. Bonferroni‐corrected post‐hoc tests indicated that applicants with a gender‐neutral request were more likely to be misgendered than both applicants with a feminine and a masculine request (see Table 2). Regarding the email to the substitute, the same pattern was found, fully supporting Hypothesis 2a.

The *willingness to engage in work‐related contact* with applicants requesting to be referred to in a gender‐neutral way appeared *higher* than for applicants with a binary‐gendered request (see Table 3), but a one‐way between‐subjects ANOVA showed that this difference was not statistically significant. However, adding the motivation to act without prejudice, *F*(2, 179) = 17.07, *p* < .001, or political orientation, *F*(2,184) = 4.21, *p* = .016, as a covariate showed statistically significant differences: Sidak‐corrected post‐hoc tests showed that participants were more willing to work with an applicant with a gender‐neutral request than with a masculine request (motivation to act without prejudice: *p* = .018; political orientation: *p* = .019; OS, Table S7), contradicting Hypothesis 2b. Applicants with a gender‐neutral request neither received significantly *shorter response emails*, nor was the *expressed positive affect* significantly lower for them, supporting neither Hypothesis 2c nor 2d.

Moreover, the results indicated descriptively highest competence and warmth perceptions for applicants with a gender‐neutral request, challenging the hypothesis that they would be perceived as less competent and warm than applicants with a masculine or feminine request, respectively. Whereas applicants with a masculine request were perceived as a bit more competent than applicants requesting to be referred to in a feminine way, and vice versa for warmth, no significant differences between applicants' *competence* or *warmth* were obtained, not supporting H3. One might suspect that participants paid too little attention or responded carelessly, but large effects were obtained regarding perceived masculinity and femininity, with applicants with the masculine request rated higher (lower) on masculinity (femininity) than applicants with the feminine request, and applicants with the gender‐neutral request in between, corroborating expectations of moderate androgyny.

### Discussion

The pre‐experiment showed that applicants requesting to be referred to in a gender‐neutral manner faced considerable misgendering, both when being addressed directly and (somewhat more) in an email to a colleague. Other than that, no hint of interpersonal discrimination was found nor formal hiring discrimination. We could also not find significant differences in perceived competence or warmth (but in masculinity and femininity). Descriptively, our participants indicated rather positive impressions of applicants requesting to be referred to in a gender‐neutral manner.

These outcomes are unexpected given evidence indicating ongoing workplace discrimination (Davidson, 2016; de Vries et al., 2020; Fütty et al., 2020; Seiler‐Ramadas et al., 2021; Waite, 2021). The lack of discrimination against nonbinary applicants in our study could be due to growing acceptance, non‐validated measures (i.e. word count and displayed affect) and to polarization on the topic of trans* rights in Germany. While most Germans support discrimination protections for LGBT individuals, support has declined since 2021 (Ipsos, 2024). Additionally, there is a noticeable global gender gap in support of trans people, with young men showing less support than women (Ipsos, 2024). This experiment's sample was predominantly composed of left‐wing, young women. Thus, a replication with a more diverse sample is indicated.

While undertaking this replication, including a direct measure of attitudes towards nonbinary genders is valuable (Molin et al., 2021), as well as an open‐ended measure capturing spontaneous stereotypes. Furthermore, perceived lack‐of‐fit should be explored.

## REVIEWED EXPERIMENT

The reviewed experiment extended the pre‐experiment in the following ways. We again tested all hypotheses of the pre‐experiment (H1: hireability, H2a: misgendering, H2b: work‐related contact); given that word count and affect have not previously been tested for emails, H2c‐d were regarded as exploratory. Again, in H3, we tested competence and warmth ascriptions. We aimed to recruit a more diverse sample and included a measure of openness towards nonbinary gender that should moderate the relationship between the request to be referred to in a gender‐neutral manner and the dependent variables (H4). Concretely, given higher openness towards nonbinary gender, we expected to replicate the null findings of the pre‐experiment, whereas with lower openness, the original hypotheses should be supported.

An additional hypothesis targeted spontaneous stereotypes. Since individuals with binary‐gendered requests are more easily assumed to be cis (cisnormativity), we assumed that individuals with a gender‐neutral request will be ascribed stereotypes similar (yet not identical) to trans people in an open question format. Therefore, we anticipated ascriptions for applicants requesting to be referred to in a gender‐neutral way to relate to normality and health more than those of applicants requesting to be referred to in a feminine or masculine way (H5). We also explored whether stereotypes reflect competence and warmth.

Lastly, as social desirability and ease of using gender‐neutral language are higher when addressing the applicant directly, we expected misgendering of applicants with a gender‐neutral request to be more likely in the email to a colleague than to the applicant (H6). Perceived lack‐of‐fit was explored.

### Method

#### Participants and design

We used a professional service (Bilendi) to recruit a diverse sample regarding gender and political orientation, targeting the German adult population (18 to retirement age of 67) with a quota for gender (50:50 ratio of women to men, plus inclusion of all nonbinary participants). As a proxy for political orientation, we used political party preference (i.e. sample proportions based on the distribution of seats in the Bundestag and 10% for non‐voters and voters with a party preference not represented there). Even though several studies suggest that recruiters and laypeople judge applicants similarly (e.g. Heilman & Okimoto, 2008; Sinclair & Agerström, 2020; Steffens, Preuß, & Scheifele, 2019) we assessed participants' experience in personnel selection using a 4‐point scale (1—*none at all* to 4—*a lot*, based on Ball et al., 2025). To ensure generalizability, we compared results in two subgroups: those with no experience (*n* = 507) and those with at least some experience (*n* = 768, 60.24%). Participants learned that passing attention checks is necessary for final payment. Again, robustness checks excluded participants who deviated more than 3 *SD* from the mean completion time (*n* = 5) or did not infer the applicant's intended gender identity correctly (total *n* = 174). As a minor adaptation, checks also excluded participants who did not remember the participant's requested form of address and pronouns correctly (total *n* = 81, exact wording: see OS, Table S3). As per the panel provider's regulations, participants who failed attention checks or skipped pages were immediately excluded. Unexpectedly, we encountered apparently fake, automated responses. Thus, we added another exclusion criterion: Open‐response data quality was assessed, with main analyses including all cases except those that were clearly inauthentic or of low quality (criteria: see OS), whereas robustness checks included only responses without any conspicuities.

Aiming for detecting small effects (ƒ = .10), with a power of 1 – β = .90 and given α = .05, the required sample size for one‐way three‐level ANOVAs is *N* = 1269. This sample size is sufficient also for subsample and moderation analyses. After excluding 11 participants who did not consent to data usage and 105 with low data quality, the final sample consisted of 1275 individuals (robustness check with all criteria: *N*_RC_ = 1000). Participants' age ranged from 18 to 67 (*M* = 47.87, *SD* = 12.81), and gender distribution was balanced (50.5% women, 48.0% men, less than 1% for each remaining self‐categorization). 29.1% selected the scale midpoint for political orientation (*M* = 5.76, *SD* = 2.13) and most participants were employed (62.35%). 17.57% reported knowing a nonbinary person, and 12.39% were not familiar with the term (scale: *1—not familiar* to *5—very familiar*; *M* = 3.35, *SD* = 1.26).

#### Procedure and measures

The procedure was identical to the pre‐experiment with the following differences (for details, Table S3). Briefly, after reading a job posting for an IT consultant position which included customer contact and an application email, participants were asked to communicate their hiring decision in a reply email and in an email to a colleague. They then judged the applicant's warmth, competence, masculinity and femininity. Moderator and covariates were assessed as well. A minor adaptation is that the identical gender‐neutral names were used in all conditions (i.e. Alex, Charly and Lou), avoiding any potential differences in name stereotypes. To understand whether misgendering was by mistake or intentional, we asked all participants after the response emails whether they would correct their language to align with the request of the applicant if they had the chance, for both emails (exact wording: see OS). We extended the scale measuring willingness to engage in work‐related contact (6 items) to improve content validity.

One major adaptation was the addition of an open‐response format as implemented by Nicolas et al. (2022, 2024) to examine *Spontaneous Stereotype Content*, allowing to detect unique stereotypes and to capture the representativeness of stereotype dimensions. Similar to Nicolas et al. (2024), participants were asked: ‘What are the first 6 characteristics or traits that spontaneously come to mind when you think about the applicant?’ (full instructions: see OS) and filled out a list of six blank spaces.

Potential moderators were collected before the manipulation for half of the participants and afterwards for the other half to ensure that they do not affect the manipulation nor are affected by it. To measure specific beliefs about nonbinary gender identities, we added 12 items of the *Openness towards Non‐Binary Gender* (ONBG) scale (Molin et al., 2021; e.g. ‘There are more than two gender categories’).

Four items by Sczesny et al. (2022) measured the *perceived fit* of the candidate for the advertised position (e.g. ‘This job will likely meet the skills and abilities of the applicant’), and we measured the *warmth/competence required for the job* using the same items as for the applicant.

#### Analysis pipeline

Misgendering in emails was manually coded and tested with chi‐square tests and Fisher's exact tests. The difference in misgendering of applicants with a gender‐neutral request between emails (H6) was analysed with a McNemar's test. For Hypotheses 2b‐d and 3, between‐subjects ANOVAs or Kruskal–Wallis tests were computed. Simple moderation analyses (linear and binomial logistic regression with robust *SEs*) for Hypothesis 4 were conducted in RStudio version 2025.9.1.401 (Posit team, 2025).

The analysis of spontaneous stereotypes (H5) used R code adapted from Nicolas et al. (2024). After preprocessing (e.g. removal of punctuation), responses were converted into text embeddings: vector representations capturing the semantic content of one or more words. We then measured the cosine similarity (i.e. semantic relatedness) between each response embedding and dimension embedding (i.e. vector representation capturing the average semantic information of the most prototypical words from a dimension) of the competence, warmth, uniqueness (for normality) and health dimensions, resulting in a cosine similarity matrix with scores theoretically ranging from −1 (exactly opposite) to 1 (exactly the same). Dimension embeddings were calculated by first translating the most prototypical words (see Table S8) into German. Similarities between responses and dimensions were averaged and used to conduct mixed model analyses (see Nicolas et al., 2022, 2024), with participants and stimuli (names) as random factors. In practical terms, high similarities indicate whether participants associate the applicant with terms representing these dimensions, but not whether responses correspond to low (e.g. ‘unhealthy’) or high (e.g. ‘healthy’) scores on those dimensions.

Perceived lack‐of‐fit was explored in supplementary path analyses using PROCESS (Hayes, 2022) (see OS). Potential covariates (e.g. political orientation) were analysed separately in supplementary logistic regressions (hireability), Firth's bias‐reduced logistic regressions (misgendering) and ANCOVAs (willingness to engage in work‐related contact, word count, displayed affect).

### Results and discussion

Assumptions about applicants' being trans* in the manipulation check showed a difference between the masculine‐request (20.58%) and feminine‐request conditions (40.78%). Moreover, there was a consistent male bias (see OS for details, Table S9).

Descriptive statistics and analyses are summarized in Tables 4 and 5. In brief, applicants with gender‐neutral (or feminine) requests faced hiring discrimination and misgendering. Type of request significantly affected *hiring decisions* (see Table 4). Post‐hoc Bonferroni‐corrected comparisons showed that applicants who asked to be addressed in a gender‐neutral way were hired less often than masculine‐request applicants (χ^2^(1, *N* = 647) = 7.39, *p*_*adj*_ = .015, *V* = .11, *OR* = 0.61), consistent with H1. Moreover, feminine‐request applicants were hired less often than masculine‐request applicants (χ^2^(1, *N* = 620) = 6.15, *p*_*adj*_ = .030, *V* = .10, *OR* = 0.63; see OS for details, including Table S10). These effects remained significant in all robustness checks, except when excluding people who miscategorized gender identity. Given the small effect size, results were not significant in subgroups: Descriptively, among participants with personnel‐selection experience, applicants in the gender‐neutral condition were hired least often, whereas their hiring rate slightly exceeded that of applicants using feminine forms among those without such experience. These findings, given the male‐typed job context, align with the lack‐of‐fit model and discrimination surveys (Frohn & Heiligers, 2024; Shannon, 2022).

**TABLE 4:** Overview of descriptives and statistical tests of the reviewed experiment for binary outcomes (hiring decisions and misgendering).

Measure	Condition				Statistical test (3 conditions)	*p*
	Gender‐neutral (*n* = 336)	Feminine (*n* = 309)	Masculine (*n* = 311)	[No request] (*n* = 319)		
Formal discrimination						
Hiring decision (H1)	67.26% (226)	67.96% (210)	77.17% (240)	79.62% (254)	χ^2^(2, *N* = 956) = 9.32	.009
Sample: no experience	71.94% (100) *n* = 139	67.54% (77) *n* = 114	80.80% (101) *n* = 125	84.50% (109) *n* = 129	χ^2^(2, *N* = 378) = 5.68	.059
Sample: at least some experience	63.96% (126) *n* = 197	68.21% (133) *n* = 195	74.73% (139) *n* = 186	76.32% (145) *n* = 190	χ^2^(2, *N* = 578) = 5.24	.073
Interpersonal discrimination						
Misgendering (H2a)						
Any email	54.17% (182)	18.18% (56)	3.86% (12)		χ^2^(2, *N* = 955) = 226.56	<.001
Excluding gendered words	37.80% (127) *n* = 336	12.01% (37) *n* = 308	3.86% (12) *n* = 311	Perceived gender identity: NB: 12.23% (39) F: 16.93% (54) M: 70.85% (226)	χ^2^(2, *N* = 955) = 136.20	<.001
Response email	25.08% (83) *n* = 331	11.73% (36) *n* = 307	1.61% (5) *n* = 310		Fisher's exact test (*N* = 948)	<.001
Excluding gendered words	22.52% (68) *n* = 302	9.71% (27) *n* = 278	1.79% (5) *n* = 280		Fisher's exact test (*N* = 860)	<.001
Email to colleague	46.13% (155)	15.26% (47)	2.57% (8)		χ^2^(2, *N* = 955) = 190.64	<.001
Excluding gendered words	27.08% (91) *n* = 336	9.74% (30) *n* = 308	2.57% (8) *n* = 311		χ^2^(2, *N* = 955) = 88.58	<.001

*Note*: Statistical tests for the three conditions with a request for specific use of pronouns/formal address (gender‐neutral, feminine, masculine). Different *n*_*s*_ are due to missings. ‘Excluding gendered words’ excludes generic masculine forms, providing stricter tests.

Abbreviations: F, feminine; M, masculine; NB, nonbinary.

**TABLE 5:** Means, standard deviations and comparison tests of the reviewed experiment for interpersonal discrimination and stereotypes.

Measure	Condition									*p*	η^2^
	Gender‐neutral (*n* = 336)		Feminine (*n* = 309)		Masculine (*n* = 311)		[No request] (*n* = 319)		Statistical test (3 conditions)		
	*M*	*SD*	*M*	*SD*	*M*	*SD*	*M*	*SD*			
Interpersonal discrimination											
Work‐related contact willingness (H2b)	4.84	1.86	4.81	1.69	4.94	1.55	5.06	1.45	*H*(2) = 0.79	.674	.00
Politeness											
Word count	38.15	23.02	37.38	19.19	34.83	18.25	35.87	17.17	*F*(2, 953) = 2.33	.098	<.01
Positive affect	11.57	5.03	12.18	4.64	10.19	4.81	10.35	4.53	*F*(2, 953) = 13.78	<.001	.03
Negative affect	0.88	1.96	0.80	1.61	0.56	1.76	0.50	1.42	*H*(2) = 11.4	.003	.01
Stereotypes (H3)											
1. Competence	4.18	0.90	4.15	0.85	4.20	0.80	4.18	0.77	*F*(2, 953) = 0.35	.703	<.01
2. Warmth	3.93	0.94	4.00	0.81	4.00	0.80	4.14	0.68	*H*(2) = 0.25	.882	.00
3. Femininity	2.90	0.82	3.32	0.94	2.06	0.97	2.55	0.88	*H*(2) = 244	<.001	.25
4. Masculinity	2.91	0.80	2.39	0.90	3.58	0.90	3.24	0.80	*F*_Welch_(2, 627.09) = 135.03	<.001	.30
Perceived fit	5.48	1.45	5.44	1.48	5.67	1.29	5.67	1.33	*H*(2) = 2.21	.332	<.01

*Note*: Statistical tests for the three conditions with a request for specific use of pronouns/formal address (gender‐neutral, feminine, masculine).

*Misgendering* occurred in line with Hypothesis 2b (see Table 4): Applicants who asked for gender‐neutral forms were misgendered more often than both applicants who requested feminine forms (χ^2^(1, *N* = 644) = 87.8, *p*_*adj*_ < .001, *V* = .37, *OR* = 5.30) and those who requested masculine forms (χ^2^(1, *N* = 647) = 192.0, *p*_*adj*_ < .001, *V* = .55, *OR* = 28.96), according to Bonferroni‐corrected post‐hoc tests. This effect persisted when misgendering was more narrowly defined via formal address and pronouns, excluding other gendered words such as generic masculine forms (feminine: χ^2^(1, *N* = 644) = 59.0, *p*_*adj*_ < .001, *V* = .30, *OR* = 4.43, masculine: χ^2^(1, *N* = 647) = 126.0, *p*_*adj*_ < .001, *V* = .41, *OR* = 14.91). Surprisingly, feminine‐request applicants were also misgendered more often than masculine‐request applicants (narrow definition: χ^2^(1, *N* = 619) = 14.7, *p*_*adj*_ < .001, *V* = .15, *OR* = 3.37). Among participants who misgendered, about a third (half in the feminine‐request condition) indicated they would not correct it (see Table S11). In line with prior studies (Jacobsen et al., 2024), the medium to large effects for gender‐neutral‐request applicants clearly indicate misgendering.

As expected (H6), applicants with a gender‐neutral request were misgendered more often in the email to a colleague than to the applicant, but only when all gendered words were included (χ^2^(1, *N* = 948) = 39.3, *p*_*adj*_ < .001, Cohen's *g* = .25, *OR* = 2.98). This suggests that ease of language use, rather than social desirability, drove the difference between email recipients.

**FIGURE 1:**
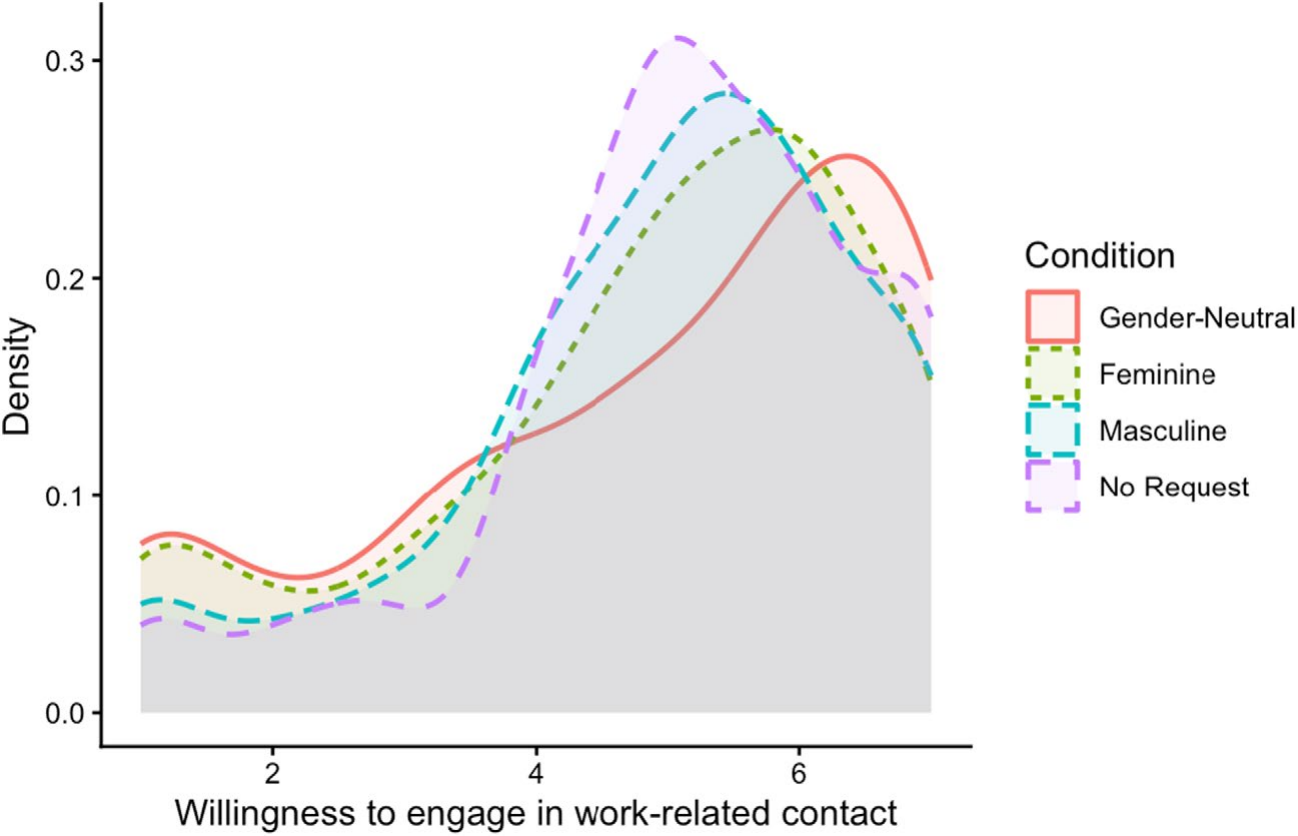
Density plot of the willingness to engage in work‐related contact by request type.

The *willingness to engage in work‐related contact* (α = .94) did not differ significantly between applicants with a gender‐neutral request and those with a binary‐gendered request, contrary to predictions (H2b, Table 5, Figure 1). Including covariates did not change these results (Tables S12–14, but see OS for moderations, Tables S15–16 and Figures S2–6). Measures of *politeness* did not support H2c and provided no or only partial support for H2d (see Table 5; detailed report see OS).

**FIGURE 2:**
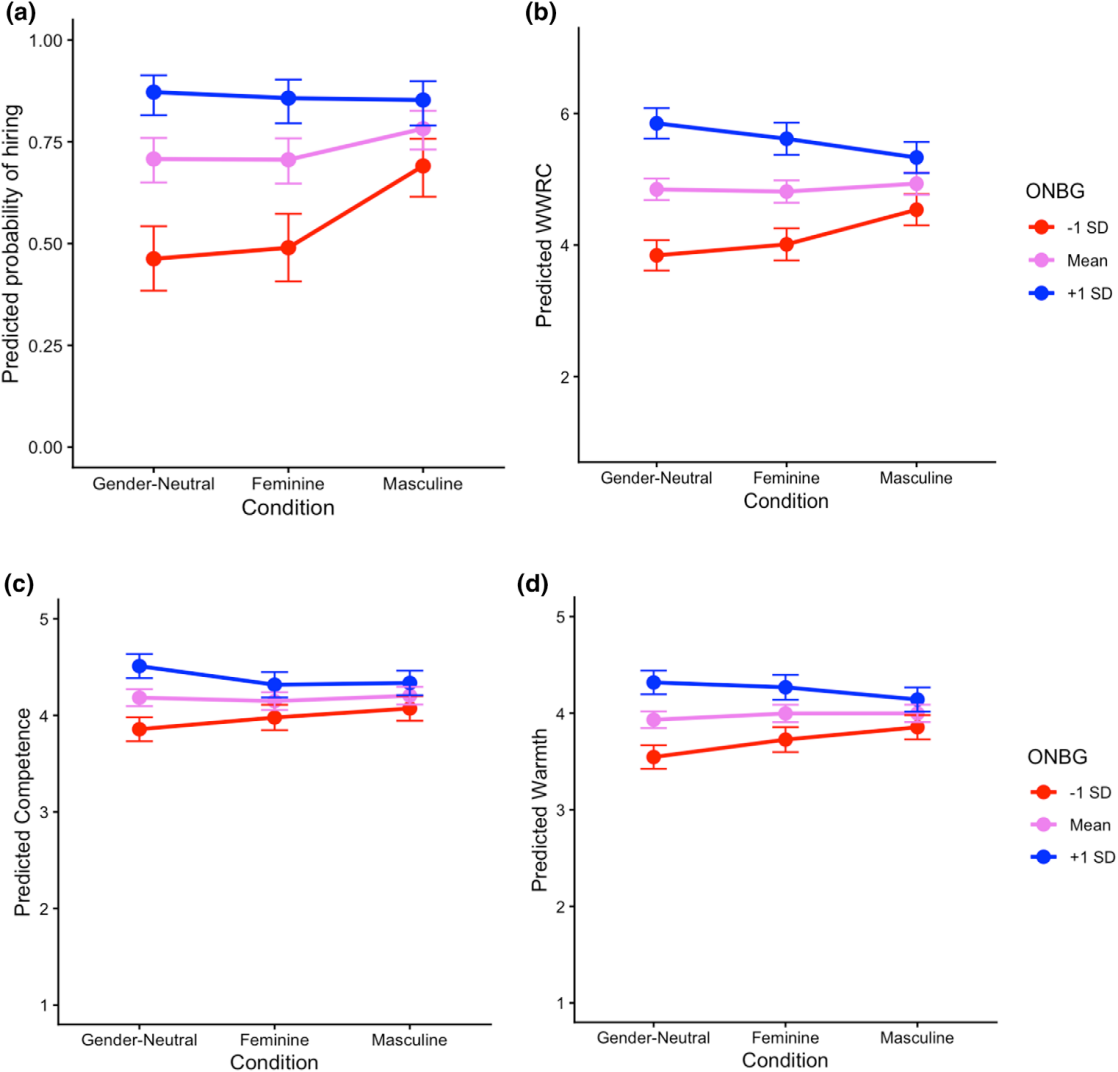
Estimated marginal means for the moderating effect of openness towards nonbinary gender (ONBG) on (a) hiring, (b) willingness to engage in work‐related contact (WWRC), (c) perceived warmth and (d) perceived competence of applicants.

In line with predictions, *openness towards nonbinary gender* (α = .96, *M* = 4.21, *SD* = 1.83; OS, Table S17) moderated the relationship between the gender‐neutral vs. masculine request and hiring, *b* = −0.31, *SE* = 0.11, *z* = −2.86, *p* = .004, *OR* = 0.74 (see Figure 2a). As expected, when ONBG was higher (+ 1 *SD*, H4a), hiring probabilities across all conditions were comparable, and when ONBG was lower (−1 *SD*, H4b), applicants with a masculine request were more likely to be hired than applicants with a gender‐neutral (and feminine) request (*b* = 0.95, *SE* = 0.24, *p* < .001, *OR* = 2.60). As shown in Figure 2b, ONBG also moderated the effect of applicant request on the willingness to engage in work‐related contact (χ^2^(2) = 23.54, *p* < .001, $\bar{R}$ ^2^_*partial*_ = .026). Again, when ONBG was low (−1 *SD*, H4b), participants were more willing to engage in contact with masculine‐request applicants than those with a gender‐neutral (or feminine) request (*b* = 0.69, *SE* = 0.19, *p* < .001, *OR* = 1.99). Contrary to expectations, when ONBG was high (+1 *SD*, H4a), contact willingness was *lower* for applicants with a masculine request than for those with a gender‐neutral request (*b* = −0.52, *SE* = 0.16, *p* = .001, *OR* = 0.59; cf. also Figure 1).

For competence and warmth, we found similar interaction patterns, apart from the feminine‐request condition (see OS and Figure 2c,d). For misgendering, we found no significant interaction with ONBG (χ^2^(2) = 0.87, *p* = .647; narrow definition: χ^2^(2) = 0.86, *p* = .651).

Corroborating H4, contact willingness (H2b) and stereotype content (H3) ratings depended on participants' ONBG. The unexpected preference for contact with applicants using gender‐neutral forms of high‐ONBG participants (H4a) potentially represents a motivation to provide support and counteract potential exclusion or reflects a history of positive intergroup contact (see Kauff et al., 2021). However, this does not explain why H3 was not supported.

Following the expected pattern of H4, the effect of request type on *perceived fit* (α = .88) was conditional on ONBG (χ^2^(2) = 11.80, *p* = .003, $\bar{R}$ ^2^_*partial*_ = .011). Therefore, we included this moderation in our mediation models on hiring (see OS, Figures S7–8). Perceived fit (more than stereotype content) mediated hiring odds, whereas job stereotypes did not moderate the relationship between applicant stereotypes and hiring, contrary to lack‐of‐fit assumptions.

We did not find evidence for H5 in the embedding analyses on *spontaneous stereotypes*: Averaged text embeddings describing applicants with a gender‐neutral request were not more strongly associated with health or normality dimensions than those of other applicants (see OS, including Table S18). However, raw data patterns indicate that these null findings should be interpreted with caution. We also explored email content beyond the preregistered hypotheses (see OS).

## GENERAL DISCUSSION

Across two experiments simulating recruitment scenarios, we examined stereotyping and discrimination of applicants who disclosed preferred forms of address and pronouns, signalling nonbinary versus binary gender identities. Participants evaluated an application email for an IT consultant position, a stereotypically masculine job with client interaction. We hypothesized that nonbinary applicants would face discrimination.

In the convenience‐sample pre‐experiment, discrimination against applicants requesting gender‐neutral language was limited to misgendering, whereas in the more representative sample, clear evidence of discrimination emerged. Applicants with a gender‐neutral request were hired less often, elicited more negative affect (see Table 5 & OS) than those requesting masculine forms, and were misgendered more often than applicants in the masculine‐ and feminine‐request conditions (supporting H1, H2a).

For participants high in openness towards nonbinary gender (ONBG), hiring odds did not differ between conditions (supporting H4a), but they showed slightly higher contact willingness and more positive evaluations of nonbinary applicants.

Participants low in ONBG showed lower hiring odds and contact willingness for nonbinary versus masculine applicants (supporting H4b) and rated nonbinary applicants lower in warmth, competence and job fit. Path analyses with ONBG as a moderator showed that perceived fit mediated hiring discrimination. No consistent differences appeared in the similarity between open‐ended characteristic descriptions and representative words for health and normality, contrary to H5. In sum, misgendering occurred in both samples, hiring discrimination emerged only in the more diverse sample, and other interpersonal discrimination indicators appeared only when considering participants' ONBG.

### Theoretical implications

The central implication of our experiment on whether people who request gender‐neutral language are discriminated in application reviews is that findings were not driven by shared stereotype content (competence and warmth; Fiske et al., 2002) but by beliefs about gender (ONBG: Molin et al., 2021) or participants' attitudes (i.e. prejudiced evaluators, Sterkens et al., 2025).

Little discrimination was visible in the convenience‐sample pre‐experiment, where ONBG was not assessed. In the larger sample, formal discrimination was more detectable than self‐reported interpersonal discrimination. Whereas motivation to act non‐prejudiced significantly moderated only self‐reported evaluations (see OS), lower ONBG also moderated formal discrimination against nonbinary compared to male applicants. Thus, research on discrimination of nonbinary targets should rely on diverse samples and methods and include relevant moderators. Experience in personnel selection, although sometimes discussed (Koch et al., 2015), was not a major factor.

Only for direct ratings of masculinity and femininity did we obtain the hypothesized pattern (moderate androgyny), with nonbinary applicants rated in between male and female applicants, consistent with work on gender and sexual‐minority stereotypes (Asbrock, 2010; Klysing et al., 2021). A lack of main effects on competence and warmth, despite a substantial sample size, fits inconsistent findings on warmth and competence (McCarty & Burt, 2024), precluding strong theoretical implications. Recent research also suggests a shift from traditional stereotype patterns (Eagly et al., 2020; Sánchez‐Rodríguez et al., 2023). Similarly, as job stereotypes did not moderate the relationship between applicant stereotypes and hiring, lack‐of‐fit based on competence and warmth did not seem to drive hiring decisions, contrary to predictions derived from theoretical models. Although perceived fit predicted hiring decisions, these perceptions depended on evaluators' ONBG. Thus, our results suggest prejudice‐based discrimination towards applicants who deviate from gender norms, in a broad sense aligning with role congruity theories, but provide little evidence that stereotype‐based fit explains the outcomes.

Open responses (see OS) revealed a recurring rejection rationale: female, and especially nonbinary applicants were depicted as disruptive, with participants raising client‐ and team‐related concerns, consistent with previous findings (Van Borm & Baert, 2018; contact denial: Fütty et al., 2020). The absence of expected spontaneous stereotypes warrants further investigation (see Limitations).

Results for female and nonbinary applicants were similar for several outcomes, which was unexpected regarding ONBG. This may be because many participants perceived female applicants as trans* too, perhaps because the gender‐neutral names were subjectively associated with men. This aligns with our observation of a male bias, consistent with prior research showing that gender‐neutral names (Renström & Klysing, 2025) and pronouns (Lindqvist et al., 2019; Renström et al., 2023) frequently default to (normative) masculine interpretations, whereas explicitly nonbinary pronouns do not. These factors complicate interpretation, but whether applicants were perceived as trans* or cis women or otherwise, the similarity of results may reflect a penalty for deviating from cisnormative or role‐specific expectations, or both.

Across both experiments, misgendering apparently reflects a lack of habitual use of gender‐inclusive language. Although left‐leaning political orientation (see OS) was associated with less misgendering, it also occurred when participants expressed no intent to do so. Still, a considerable portion of participants also misgendered intentionally (Table S11), perhaps in response to perceived threats to the gender/sex binary (Morgenroth & Ryan, 2021) or masculinity (e.g. Lorenz et al., 2026).

Incidentally, we found evidence of androcentrism: the applicant without pronoun request was most often assumed to be male and received the most favourable outcomes. This suggests that some participants generally hold negative attitudes towards pronoun requests.

### Practical implications

Attitudinal variability suggests that educational efforts fostering greater ONBG could partly mitigate discrimination and, more broadly, alleviate conflict concerns. Attitude change can be facilitated by creating structured opportunities (e.g. intergroup contact) to consider the experiences of nonbinary, trans and gender nonconforming individuals and by normalizing inclusive norms in interactions and communications. Misgendering, often unintentional but consequential (Corby et al., 2025), highlights the need for training habitual use of inclusive language.

Binary‐gendered people rarely need to indicate pronouns. However, using gender‐neutral/gender‐expansive linguistic gender expressions or having a name, appearance or voice not matching typical expectations creates a practical need to do so, placing applicants at a disadvantage even without coming out or being nonbinary. To mitigate this, hiring practices could default to gender‐inclusive language in communication (e.g. using names instead of ‘Mr.’ or ‘Ms.’). Furthermore, organizations could update demographic questions and communication practices to avoid unnecessary binary classifications, which are problematic for gender‐diverse individuals (Klysing & Steffens, 2025).

### Strengths, limitations and future research directions

Our study combined self‐report measures with behavioural measures, such as simulated hiring decisions, misgendering and content analysis, providing a robust assessment of discrimination. Because our manipulation of gender was subtle, stronger effects might emerge with explicit gender information. Furthermore, since participants were required to write response emails, we could not examine differences in response rates (see McCarty, 2024).

Our analysis using text embeddings has limitations. Because the study was conducted in German, we relied on only one of several methods (Nicolas et al., 2022, 2024) for spontaneous stereotypes, and cosine similarities may have failed to detect differences due to noise in open‐ended responses, overlaps between dimension, diluted meanings when averaging embeddings or pretrained models not fully capturing the hiring‐specific context. Combining embeddings with dictionary‐based counts (Nicolas et al., 2021) could result as more informative.

Since gender‐neutral requests were often interpreted as male and the treatment of nonbinary individuals may vary by assigned gender at birth (Davidson, 2016; Dray et al., 2020; Shannon, 2022), future research should examine potential differences associated with perceived gender/sex background. It is further valuable to investigate female‐typed jobs to explore whether evaluation patterns depend on job gender‐typing and consider whether participants judge fit based on how socially ‘appropriate’ individuals are perceived to be for the role (e.g. age appropriateness in teaching: Landry & Yacknovets, 2022). Given the importance of ONBG as a moderator, future work could investigate potential predictors of ONBG, such as positive contact (see Kauff et al., 2021). ONBG's distinction from related concepts and its role within the broader gender system also warrant examination.

### Conclusion

Hiring discrimination against nonbinary (and female) applicants was observed only in a large, diverse sample, highlighting the need for such samples in research on nonbinary individuals and explaining discrepancies between discrimination experiences indicated in surveys and positive assessments observed in convenience samples. Most forms of interpersonal discrimination, including lower willingness for work‐related contact and more negative evaluations, were observed only in participants with low openness towards nonbinary gender, suggesting that beliefs or negative attitudes, rather than shared stereotypes of warmth and competence, drive these effects. Misgendering occurred across experiments, reflecting unintentional behaviour in some and deliberate mistreatment in other cases. Our findings emphasize the need to address both biases and habitual language practices to reduce barriers for nonbinary applicants.

## AUTHOR CONTRIBUTIONS

**Lou Dörr:** Conceptualization; methodology; data curation; formal analysis; investigation; project administration (Stage 2); writing – original draft; writing ‐ review and editing; visualization; validation. **Elena Ball:** Conceptualization (Stage 1); methodology (Stage 1); formal analysis (Stage 1); investigation (Stage 1); writing – review and editing; project administration (Stage 1); validation (Stage 1). **Pia Vogel:** Conceptualization (Stage 1); data curation (Stage 1); formal analysis (Stage 1); investigation (Stage 1); methodology (Stage 1); writing ‐ original draft (Stage 1); writing – review and editing; validation (Stage 1). **Claudia Niedlich:** Conceptualization; methodology; project administration; resources; supervision; writing – review and editing; funding acquisition; validation. **Melanie C. Steffens:** Conceptualization; methodology; supervision; writing – original draft (Stage 2); writing – review and editing; resources; funding acquisition; validation.

## CONFLICT OF INTEREST STATEMENT

The authors declare that they have no conflict of interest.

## ETHICS STATEMENT

The present research was approved by the Local Ethics Committee of the Faculty of Psychology, RPTU University Kaiserslautern‐Landau. Participants were treated following APA standards and the Declaration of Helsinki. Data were collected anonymously; participants were fully debriefed and decided whether they allow us to analyse their data or not after data collection. Informed consent was obtained at the beginning of data collection.

## Supporting information


Data S1.

## Data Availability

Pre‐experiment preregistration and data are available at https://researchbox.org/3411. The approved Stage 1 protocol of the reviewed experiment, data and online supplement can be found at https://osf.io/8hmec/overview?view_only=fe3a60f3e1fc45c7a186947ac15a8ace.

## Footnotes

^1^
The asterisk is used to acknowledge the diversity of experiences and identities (Stryker, 2017). In this article, the absence of an asterisk indicates reference to binary trans experiences.
