# Supplementary material for: gender.neutral@work.de: An experimental approach to the discrimination of nonbinary individuals during job applications
Source: Br J Soc Psychol. 2026 Apr 28;65:e70081. doi: 10.1111/bjso.70081 (PMC13123307; doi:10.1111/bjso.70081)
Supplement: Supplementary file 1 — Data S1. [file BJSO-65-0-s001.docx]

**Online Supplement**

[Openness Towards Nonbinary Gender: Pre-Study Results 2](#_Toc215145200)

[Theoretical Schematic 3](#_Toc215145201)

[Juxtaposition of Preregistration and Pre-Experiment Hypotheses 3](#_Toc215145202)

[Comparison of Pre-Experiment and Reviewed Experiment Hypotheses and Methods 5](#_Toc215145203)

[Pre-Experiment: Additional Demographics 7](#_Toc215145204)

[Pre-Experiment: Robustness Checks 8](#_Toc215145205)

[Manipulation Wording 8](#_Toc215145206)

[Pre-Experiment: Analyses Including the Fourth Experimental Condition 9](#_Toc215145207)

[Job Posting (Translated) 10](#_Toc215145208)

[Pre-Experiment: Supplementary Variables 10](#_Toc215145209)

[Processing Fluency 11](#_Toc215145210)

[Dehumanization 12](#_Toc215145211)

[Gender Essentialism 12](#_Toc215145212)

[Motivation to Act Without Prejudice 12](#_Toc215145213)

[Political Orientation 13](#_Toc215145214)

[Reviewed Experiment Methods 14](#_Toc215145215)

[Instructions for Spontaneous Stereotypes 14](#_Toc215145216)

[Prototypical Words for Dimension Embeddings 15](#_Toc215145217)

[Exclusion Criteria Based on Open-Response Data Quality 15](#_Toc215145218)

[Reviewed Experiment Analyses 15](#_Toc215145219)

[Perception of Applicant’s Sex/Gender & Transness 15](#_Toc215145220)

[Detailed View on Hiring Discrimination (Not Registered) 16](#_Toc215145221)

[Misgendering Intention 18](#_Toc215145222)

[Politeness in Response Emails 18](#_Toc215145223)

[Covariates 20](#_Toc215145224)

[Main Effects Across Conditions 20](#_Toc215145225)

[Exploratory Moderation Analyses (Not Registered) 23](#_Toc215145226)

[Openness Towards Nonbinary Gender (ONBG) 30](#_Toc215145227)

[Main Effects Across Conditions 30](#_Toc215145228)

[Moderation of ONBG on Competence, Warmth and Perceived Fit 30](#_Toc215145229)

[Mediation Analyses 32](#_Toc215145230)

[Spontaneous Stereotypes 34](#_Toc215145231)

[Text Embedding Analyses (H5) 34](#_Toc215145232)

[Raw Data Patterns of Spontaneous Stereotypes 36](#_Toc215145233)

[Content of Emails (Not Registered) 36](#_Toc215145234)

# Openness Towards Nonbinary Gender: Pre-Study Results

| **Table S1**  *Predictors for Support of Equality Policies and Political Solidarity with Nonbinary People* | | | | | | | | | |
| --- | --- | --- | --- | --- | --- | --- | --- | --- | --- |
| Predictor | Support of equality policies  (Self-Determination Act, SBGG) | | | |  | Political solidarity with nonbinary people | | | |
|  | *B* | *SE* | β | *p* |  | *B* | *SE* | β | *p* |
| Intercept | 3.44 | 0.49 | .02 | < .001 |  | 3.06 | 0.85 | .01 | < .001 |
| Openness towards nonbinary gender (ONBG) | 0.37 | 0.05 | .52 | < .001 |  | 0.51 | 0.09 | .45 | < .001 |
| (Negative) attitudes toward nonbinary individuals | -0.30 | 0.07 | -.34 | < .001 |  | -0.17 | 0.09 | -.13 | .058 |
| Transphobia | 0.03 | 0.06 | .04 | .612 |  | -0.13 | 0.09 | -.12 | .176 |
| Biological theory of gender | -0.00 | 0.04 | -.00 | .987 |  | -0.06 | 0.06 | -.04 | .301 |
| Social theory of gender | -0.04 | 0.05 | -.05 | .449 |  | 0.16 | 0.07 | .12 | .028 |
| Modern sexism | -0.07 | 0.05 | -.07 | .182 |  | -0.14 | 0.07 | -.10 | .042 |
| Normative gender role orientation | -0.04 | 0.06 | -.03 | .489 |  | -0.04 | 0.08 | -.02 | .618 |
| Impression management | 0.14 | 0.06 | .08 | .019 |  | 0.13 | 0.09 | .04 | .120 |
| Participant gender | -0.03 | 0.09 | -.03 | .752 |  | -0.01 | 0.12 | -.01 | .908 |
| Political orientation | -0.04 | 0.04 | -.06 | .250 |  | -0.12 | 0.05 | -.11 | .026 |
| *Note.* Support of equality policies: *N* = 288, *R*^2^ = .70, *F*(10, 275) = 67.38, *p* < .001, Δ*R*^2^_ONBG_ = .06  Political solidarity with nonbinary people (robust *SE*s): *N* = 288, *R*^2^ = .80, *F*(10, 275) = 115.7, *p* < .001, Δ*R*^2^_ONBG_ = .05.  Δ*R*^2^_ONBG_ represents the increase in R^2^ compared to the same model without ONBG. | | | | | | | | | |

#

# Theoretical Schematic

**Figure S1**

*Theoretical Working Model*


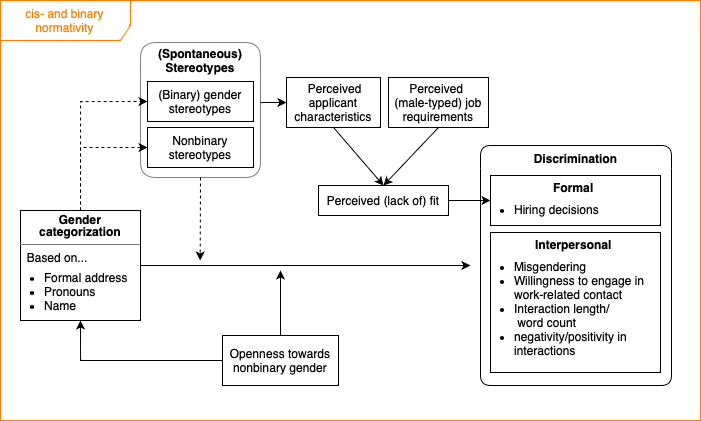


*Note.* This figure describes the roles of stereotypes, the openness towards nonbinary gender and the motivation to act without prejudice in gender-based discrimination. It differentiates the different forms of discrimination. The influences of cis- and binary normativity permeate the societal framework in which discrimination takes place and especially influences gender categorization, stereotypes and misgendering. Dashed arrows show potential influences.

# Juxtaposition of Preregistration and Pre-Experiment Hypotheses

| **Table S2**  *Juxtaposition of Hypotheses as Presented in the Preregistration in Comparison to the Manuscript for the Pre-Experiment* | | | | |  |
| --- | --- | --- | --- | --- | --- |
|  | **Preregistration** |  | | **Manuscript** |  |
| **Pre-Experiment** | | | | |  |
| *Main Hypotheses* | | | | |  |
| **1a** | Gender-ambiguous applicants will be chosen less often for a job than clearly gendered applicants. | **1** | | Applicants requesting to be referred to in a gender-neutral way will be hired less often for the male-typed job than applicants requesting to be referred to in a masculine way. |  |
| **1b** | Gender-ambiguous applicants will be misgendered more often in emails written to/about them than clearly gendered applicants. | **2a** | | Applicants requesting to be referred to in a gender-neutral way will be misgendered more often […] than applicants with a binary-gendered request. |  |
| **2** | Gender-ambiguous applicants will receive respectively lower ratings on competence and warmth in comparison to clearly gendered applicants. | **3** | | Applicants who request to be referred to in a gender-neutral way are rated lower in competence compared to those with a masculine request, and lower in warmth compared to those with a feminine request. |  |
| **3a** | Willingness to work together will be lower for gender-ambiguous applicants than for clearly gendered applicants. | **2b** | | Applicants requesting to be referred to in a gender-neutral way will […] be met with a lower willingness to engage in work-related contact […] than applicants with a binary-gendered request. |  |
| **3b** | Word count in response emails will be lower for gender-ambiguous applicants than for clearly gendered applicants. | **2c** | | Applicants requesting to be referred to in a gender-neutral way will […] be treated less politely (i.e., response emails being shorter […]) than applicants with a binary-gendered request. |  |
| **3c** | Positive affect in response emails will be lower for gender-ambiguous applicants than for clearly gendered applicants. | **2d** | | Applicants requesting to be referred to in a gender-neutral way will be treated less politely (i.e., response emails […] showing less positive affect) than applicants with a binary-gendered request. |  |
| *Exploratory Hypotheses* | | | | | |
| **4** | We explore differences in fluency between gender-ambiguous and clearly gendered applicants via between-subject ANOVAs, expecting to find lower fluency (i.e., longer writing time) for gender-ambiguous than for clearly gendered applicants. | |  | Hypothesis not included in manuscript (but see “Supplementary Variables” in this Online Supplement). |  |
| **5** | We explore via linear and binomial regression analyses whether there is an influence of participants' political orientation or their motivation to respond without prejudice on competence and warmth, willingness to work together, humanization, and selection decision. | |  | Reported in manuscript for the main hypotheses when relevant (2b) and in the Online Supplement for the exploratory hypotheses (see “Supplementary Variables”) when relevant. |  |
| **6a** | We explore whether gender-ambiguous applicants will be dehumanized more in the recruitment process than clearly gendered applicants (female/male). | |  | Hypothesis not included in manuscript (but see “Supplementary Variables” in this Online Supplement). |  |
| **6a** | We explore via mediation analyses whether humanization and fit to gender-schemas of gender-ambiguous applicants influence competence and warmth ratings and willingness to work together. | |  | Hypothesis not included in manuscript (but see “Supplementary Variables” in this Online Supplement). |  |
| **8** | We explore via mediation analyses whether gender essentialism influences competence and warmth ratings, dehumanization, and willingness to work together for gender-ambiguous applicants. | |  | Hypothesis not included in manuscript (but see “Supplementary Variables” in this Online Supplement). |  |
| **7** | We explore whether patterns of results change when comparing gender-ambiguous applicants who mention vs. not mention their preferred pronouns/address. | |  | Hypothesis not included in manuscript (but see “Analyses Including the Fourth Experimental Condition” in this Online Supplement). |  |
|  | | | | |  |

# Comparison of Pre-Experiment and Reviewed Experiment Hypotheses and Methods

| **Table S3**  *Comparison of the Methods Used in the Pre-Experiment and the Reviewed Experiment* | |
| --- | --- |
| **Pre-Experiment** | **Reviewed Experiment** |
| *Hypotheses* | |
| *H1. Formal discrimination:*  Applicants requesting to be referred to in a gender-neutral way will be hired less often for the male-typed job than applicants requesting to be referred to in a masculine way. | Same as in the pre-experiment. |
| *Interpersonal discrimination:*  Applicants requesting to be referred to in a gender-neutral way will be  (H2a) misgendered more often and  (H2b) met with a lower willingness to engage in work-related contact.  Extending existing findings on face-to-face discrimination to emails, we also expected them to be treated less politely (i.e., response emails being shorter, H2c, and showing less positive affect, H2d) than applicants with a binary-gendered request. | H2. *Interpersonal discrimination:*  Applicants requesting to be referred to in a gender-neutral way will be…   1. misgendered more often 2. met with a lower willingness to engage in work-related contact   H6. We expect misgendering of applicants with a gender-neutral request to be more likely in the email to a colleague than to the applicant. |
| *Stereotypes:*  Applicants who request to be referred to in a gender-neutral way are rated lower in competence compared to those with a masculine request, and lower in warmth compared to those with a feminine request. | H3. *Stereotypes:*  Same as in the pre-experiment.  H5. *Spontaneous Stereotypes:*  We anticipate ascriptions for applicants requesting to be referred to in a gender-neutral way to be more similar to words representing   1. normality and 2. health   than those of applicants requesting to be referred to in a feminine or masculine way. |
|  | H4. Openness towards nonbinary gender should moderate the relationship between the request to be referred to in a gender-neutral manner and the dependent variables.  Concretely,   1. given higher openness towards nonbinary gender, we expect to replicate the null findings of the pre-experiment, whereas 2. with lower openness, the original hypotheses should be supported. |
| *Method and Main Measures* | |
| *Manipulation (between-subjects):*  Alexandra/Carla/Louisa, she/her, Ms.  Alexander/Carl/Louis, he/him, Mr.  Alex/Charly/Lou, no pronouns, Name | *Manipulation (between-subjects):*  Alex/Charly/Lou, she/her, Ms.  Alex/Charly/Lou, he/him, Mr.  Alex/Charly/Lou, no pronouns, Name  Request will be made more visible. |
| *Formal Discrimination:*   - Hiring decision | Same as in the pre-experiment. |
| *Interpersonal Discrimination*:   - Misgendering - Willingness to engage in work-related contact (3 Items) - Word count - Positive affect | *Interpersonal Discrimination*:   - Misgendering - Willingness to engage in work-related contact (6 Items) |
| *Stereotypes:*   - Warmth, Competence - Femininity, Masculinity | *Stereotypes:*  Same as in the pre-experiment.   - + Spontaneous Stereotypes |
|  | *+ Moderator:* ONBG (12 Items) |
| *Exploratory Measures* | |
| *Manipulation:*  Control: No request, neutral name | Same as in the pre-experiment. |
|  | *Interpersonal Discrimination:*   - Word count - Positive affect - + Negative affect |
| *Mechanisms:*   - Dehumanization - Cognitive Fluency | *Mechanisms:*   - Subjective Fit (Mediator) - Perceived competence/warmth of the job position (IT consultant) |
| *Covariates:*   - Motivation to act without prejudice - Gender Essentialism - Political Orientation - Gender | *Covariates:*   - Motivation to act without prejudice - Political Orientation - Gender |
| *Quality checks* | |
| The manipulation checks included questions about the applicant’s gender/sex, gender identity, transness, and non-usage of pronouns because of political reasons. | Same manipulation checks as in the pre-experiment.  + “What form of address and pronouns did the person who applied want to be addressed with? (Ms. Müller, she/her; Mr. Müller, he/him; gender-neutral form of address, no pronouns; no information was provided; I don't know)“ |
| Participants were asked how familiar they were with the term nonbinary gender (scale from 1 – “not familiar” to 5 – “very familiar”), and whether they knew any nonbinary people. | Same as in the pre-experiment.  *+ Behavioral Intent*  “Would you like to adjust the gendered language in the email to the applicant to match their request, if it doesn't already?”  “Would you like to adjust the gendered language in the email to your substitute to match the applicant's request, if it doesn't already?” |
| One attention check | Two attention checks |

# Pre-Experiment: Additional Demographics

The majority of respondents stated that they were studying or employed (55 % and 35 %). The average socio-economic status in the sample was higher than the midpoint (*M* = 6.83, *SD* = 1.31). When asked about their familiarity with the term nonbinary gender, participants responded on average with familiarity rather than unfamiliarity, *M* = 4.10, *SD* = 1.07. Table S4 provides an overview of demographics.

| **Table S4** |  |  | |  | |  | |  | |  | |  | |  | |
| --- | --- | --- | --- | --- | --- | --- | --- | --- | --- | --- | --- | --- | --- | --- | --- |
| *Pre-Experiment Demographics (Metric Variables)* | | | | | | | | | |  | |  | |  | |
| Variable | *M* | | *SD* | | median | | min | | max | | skew | | kurtosis | | *SE* |
| Age^a^ | 30.32 | | 10.83 | | 19 | | 19 | | 70 | | 1.89 | | 2.86 | | 0.69 |
| Socioeconomic status^b^ | 6.83 | | 1.31 | | 3 | | 3 | | 10 | | -0.33 | | 0.01 | | 0.08 |
| Political orientation^a^ | 3.83 | | 1.97 | | 1 | | 1 | | 11 | | 0.76 | | 0.35 | | 0.13 |
| Familiarity with term nonbinary gender | 4.10 | | 1.07 | | 4 | | 1 | | 5 | | -1.35 | | 1.36 | | 0.07 |
| *Note.* ^a^ *N* = 248, ^b^ *N* = 247 | | | | | | | | | | | | | | | |

# Pre-Experiment: Robustness Checks

Excluding participants who did not infer the intended gender of the applicant (*n* = 45) led to the same patterns of results: Only Hypothesis 2a (misgendering) was supported. One difference that emerged was that, contrary to Hypothesis 2b, willing to engage in work-related contact was significantly higher for applicants with a gender-neutral request than for applicants with a feminine (*p* = .009) and masculine request (*p* = .026) as indicated by post hoc tests using Bonferroni correction following the ANOVA, *F*(2, 140) = 5.11, *p* = .007,
η^2^ = .07. Another difference we found via Bonferroni-corrected post hoc tests after a significant ANOVA, *F*(2, 140) = 3.13, *p* = .047, η^2^ = .04, was that positive affect in the response email was significantly higher (*p* = .041) for applicants with a feminine rather than masculine request, with applicants with a gender-neutral request not significantly differing from either. This finding does not provide support for our hypothesis. Excluding participants who failed the attention check (*n* = 4) or participants who deviated more than three standard deviations from the mean time taken to finish the study (*n* = 1) each led to the same patterns of results and only Hypothesis 2a was supported.

# Manipulation Wording

The applicant’s statement on pronouns/address was put at the end of the job application:

“P.S.: Please do not use pronouns for me (neither she nor he) but gender-neutral forms of address such as "Good day, Lou Müller" for me” in the gender-neutral request condition,

“P.S.: Please use she/her pronouns and feminine forms of address such as "Good day, Mrs. Müller" for me” in the feminine request condition, and

“P.S.: Please use he/him pronouns and masculine forms of address such as "Good day, Mr. Müller" for me” in the masculine request condition.

# Pre-Experiment: Analyses Including the Fourth Experimental Condition

We also explored patterns of results for the main hypotheses for applicants without a request on their address/pronouns. No significant differences regarding the main dependent variables were found between applicants with a gender-neutral request and applicants without a request.

However, as exploratory analyses using two-sided exact Fisher’s tests show, significant differences were observed between the two conditions in terms of how they were addressed/referred to: Applicants with a gender-neutral request were more likely to be addressed and referred to in gender-neutral ways, whereas applicants with no request were more likely to be addressed in clearly gendered ways in both the response email (*p* < .001, *OR* = NA, *RR* = Inf) and the email to the substitute (*p* < .001, *OR* = NA, *RR* = .32).

We conducted analyses collapsing the two gender-neutral conditions to examine whether implied gender identity affects the dependent variables, in the sense that men or women receive gendered competence and warmth ratings and participants would be more willing to engage in work-related contact with them or hire them compared to nonbinary applicants. We found no effects on competence, warmth, and hiring decision. For willingness to engage in work-related contact, we found differences between conditions, *F*(2, 124) = 5.78, *p* = .004, η^2^ = .09. Using Bonferroni correction, post hoc tests revealed that participants were more willing to engage in work-related contact with nonbinary applicants (*p* = .008) or women (*p* = .029) than men.

# Job Posting (Translated)

**IT-consulting**

We are looking for immediate reinforcement for our IT-consulting team.

**Your tasks**

- On-site consulting services and customer support
- Assessment and improvement of companies’ IT infrastructure
- Development of customized solutions for technical challenges

**Your profile**

- Degree in computer science, business informatics or a related field
- Analytical thinking and problem-solving skills
- Customer-oriented mindset
- Experience in IT-consulting or a similar position

We look forward to receiving your application!

# Pre-Experiment: Supplementary Variables

Perceived *fluency* while writing each of the emails was assessed on a rating scale from 1 – *not easy at all* to 7 – *very easy* (“How easy was it for you to write the email to the applicant/your substitute?”). As another measure of fluency, we used the time taken to reply to each of the emails (with a longer writing time indicating lower fluency or more diligence).

*Dehumanization* was assessed via an *anthropomorphism scale* with five items from Waytz et al. (2010, e.g., "To what extent does the applicant have a mind of its own?", α = .91) and seven items on *humanization* adapted from Bastian and Haslam (2010, e.g., “I feel like this person is open minded, and can think clearly about things”, α = .86) with ratings ranging from 1 – *not at all* to 7 – *very much*.

To assess *gender essentialism*, five items from Coleman and Hong (2008, e.g., “The innate properties of a person’s gender determine what the person is like”) and eight items adapted from Morgenroth et al. (2021, e.g., “Gender identity is a category with clear boundaries: If you belong to one category, you don’t belong to another category.”) were combined and rated on a scale from 1 – *strongly disagree*" to 7 – *strongly agree* (α = .86).

*Motivation to act without prejudice* was measured using six items answered on a rating scale from 1 – *I do not agree at all* to 5 – *I totally agree* (Banse & Gawronski, 2003, e.g., “If I have a prejudiced thought or feeling, I keep it to myself”, α = .74).

Table S5 provides an overview of the means of all supplementary variables per experimental condition.

| **Table S5** | | | | | | | | | | |
| --- | --- | --- | --- | --- | --- | --- | --- | --- | --- | --- |
| *Pre-Experiment: Means and Standard Deviations of Measures Across Conditions* | | | | | | | | | | |
|  | | | Condition: Applicant request | | | | | | | |
|  | Overall |  | Gender-neutral | | Feminine | | Masculine | | No request | |
| Measure | *M* | *SD* | *M* | *SD* | *M* | *SD* | *M* | *SD* | *M* | *SD* |
| Self-reported fluency (response email) | 5.38 | 1.60 | 5.22 | 1.57 | 5.48 | 1.36 | 5.37 | 1.84 | 5.47 | 1.57 |
| Self-reported fluency (substitute email) | 5.76 | 1.49 | 5.36 | 1.68 | 5.86 | 1.23 | 5.98 | 1.48 | 5.87 | 1.43 |
| Fluency time (response email) | 286.61 | 660.85 | 426.15 | 1178.94 | 227.27 | 161.42 | 216.66 | 183.08 | 261.97 | 425.94 |
| Fluency time (substitute email) | 164.70 | 429.70 | 143.90 | 89.33 | 263.16 | 871.10 | 143.14 | 190.32 | 119.40 | 71.53 |
| Motivation to act without prejudice | 4.10 | 0.50 | 4.11 | 0.44 | 3.99 | 0.55 | 4.19 | 0.52 | 4.11 | 0.50 |
| Anthropomorphism | 5.81 | 1.15 | 6.15 | 0.97 | 5.74 | 1.21 | 5.65 | 1.22 | 5.68 | 1.17 |
| Humanness | 5.38 | 0.96 | 5.48 | 1.07 | 5.44 | 0.88 | 5.24 | 0.97 | 5.37 | 0.91 |
| Gender schema: femininity | 2.85 | 0.88 | 2.90 | 0.68 | 3.64 | 0.62 | 2.20 | 0.85 | 2.77 | 0.72 |
| Gender schema: masculinity | 3.04 | 0.82 | 2.91 | 0.54 | 2.32 | 0.66 | 3.68 | 0.79 | 3.17 | 0.67 |
| Gender essentialism | 2.95 | 1.02 | 2.95 | 0.88 | 3.09 | 1.08 | 2.89 | 1.13 | 2.90 | 0.99 |

### Processing Fluency

We explored differences in fluency between applicants with a gender-neutral and applicants with binary-gendered requests using ANOVA, expecting to find lower fluency (lower perceived fluency/longer time spent writing the emails) for applicants with a gender-neutral request. No significant differences between the conditions were found for self-reported fluency in the response email (*F*(2, 185) = 0.40, *p* = .673) as well as for response time (*F*(2, 185) = 1.76, *p* = .176) in this and in the substitute email (*F*(2, 185) = 1.17, *p* = .313). Significant differences were found regarding self-reported fluency in the substitute email (*F*(2, 185) = 3.25, *p* = .041, η^2^ = .04), with Bonferroni-corrected post hoc tests showing that self-reported fluency in the email to the substitute was significantly higher for applicants with a masculine request than for applicants with a gender-neutral request, *p* = .049.

### Dehumanization

| **Table S6** |  |  |  |  |  |  |
| --- | --- | --- | --- | --- | --- | --- |
| *Pre-Experiment: Mediation Analyses with Applicant Request as Dummy-Coded Predictor (Feminine, Masculine) and Gender Essentialism as Mediator* | | | | | | |
| Criterion | Indirect effect (feminine) | *SE* (feminine) | CI_95_ (feminine) | Indirect effect (masculine) | *SE* (masculine) | CI_95_ (masculine) |
| Competence | -0.03 | 0.03 | -0.10, 0.03 | 0.01 | 0.03 | -0.05, 0.08 |
| Warmth | -0.02 | 0.03 | -0.09, 0.02 | 0.00 | 0.02 | -0.04, 0.06 |
| WWRC | -0.06 | 0.07 | -0.22, 0.06 | 0.03 | 0.07 | -0.12, 0.17 |
| Humanness | -0.03 | 0.04 | -0.13, 0.03 | 0.01 | 0.04 | -0.06, 0.08 |
| Anthropomorphism | -0.04 | 0.05 | -0.16, 0.03 | 0.02 | 0.04 | -0.06, 0.11 |
| *Note. N* = 188. CI_95_ = 95% Confidence Interval. WWRC: Willingness to engage in work-related contact | | | | | | |

Based on findings about genderless individuals (Study 8 in Martin & Mason, 2022), we hypothesized that applicants with a gender-neutral request would be dehumanized more than applicants with binary-gendered requests and analyzed this using ANOVAs. No signifi-cant differences between conditions were found for humanness, *F*(2, 185) = 1.05, *p* = .352. Significant differences were found for anthropomorphism, *F*(2, 185) = 3.25, *p* = .041,
η^2^ = .04. Bonferroni-corrected post hoc tests revealed that the difference between applicants with a gender-neutral request and applicants with a masculine request was significant, with applicants with a gender-neutral request receiving higher ratings on the anthropomorphism scale than those with a masculine request, *p* = .036. This finding is contrary to our hypothesis.

### Gender Essentialism

We explored via mediation analyses whether gender essentialism mediates effects of applicant request on competence and warmth ratings, willing to engage in work-related contact, and humanization. Details for the indirect effects can be found in Table S6. No mediating effects were found.

### Motivation to Act Without Prejudice

We conducted our main analyses again while controlling for the influence of motivation to act without prejudice or political orientation to assess whether result patterns change (see Table S7).

Motivation to act without prejudice significantly affected responses regarding the selection decision, misgendering in the email to the substitute, competence, warmth, willing to engage in work-related contact, but not regarding misgendering, word count, and positive affect, all in the response email. Differences between conditions regarding misgendering remained after adjusting for motivation to act without prejudice, *F*(2, 179) = 17.07, *p* < .001. As Sidak-corrected post hoc tests show, applicants with a gender-neutral request were still correctly referenced less often than applicants with a feminine (*p* < .001) and masculine request (*p* < .001). After adjustment for motivation to act without prejudice, there was also a statistically significant difference in willingness to engage in work-related contact between conditions, *F*(2, 184) = 3.87, *p* = .023. As Sidak-corrected post hoc tests revealed, participants were more willing to work together with applicants with a gender-neutral request than applicants with a masculine request, *p* = .018. For all other dependent variables, motivation to act without prejudice did not change or lead to significant results regarding our hypotheses. Overall, result patterns for all main hypotheses remained the same.

### Political Orientation

Political orientation significantly affected responses regarding misgendering in the email to the substitute, competence, warmth, and willing to engage in work-related contact but not regarding the selection decision and misgendering, word count, and positive affect in the response email (see Table S7). Differences between conditions regarding misgendering remained after adjusting for political orientation, *F*(2, 179) = 16.14, *p* < .001. As Sidak-corrected post hoc tests show, applicants with a gender-neutral request were still correctly referenced less often than those with a feminine (*p* < .001) or masculine request (*p* < .001). After adjustment for political orientation, there were also statistically significant differences in the willingness to engage in work-related contact between conditions, *F*(2, 184) = 4.21,
*p* = .016. As Sidak-corrected post hoc tests revealed, participants were more willing to work together with applicants with a gender-neutral request than applicants with a masculine request (*p* = .019) but not feminine request. For all other dependent variables, political orientation did not change or lead to significant results regarding our hypotheses. Overall, result patterns for all main hypotheses remained the same.

We found that political orientation and motivation to act without prejudice each significantly influenced competence, warmth, and willing to engage in work-related contact, but not the hiring decision. A more conservative political orientation was associated with lower ratings on competence, warmth, or willing to engage in work-related contact. A higher motivation to act without prejudice was associated with higher ratings on competence, warmth, and willing to engage in work-related contact.

| **Table S7** |  |  |  |  |
| --- | --- | --- | --- | --- |
| *Pre-Experiment: Regression Analyses for Political Orientation and Motivation to Act Without Prejudice* | | | | |
|  | *B* | *SE* | β | *p* |
| Political orientation | |  |  |  |
| Competence | -0.06 | 0.03 | -.16 | .030 |
| Warmth | -0.06 | 0.02 | -.18 | .014 |
| Willing to engage in work-related contact | -0.19 | 0.05 | -.28 | <.001 |
| Hiring decision | -0.09 | 0.08 | -.18 | .282 |
| Motivation to act without prejudice | | | |  |
| Competence | 0.50 | 0.11 | .32 | <.001 |
| Warmth | 0.36 | 0.10 | .26 | <.001 |
| Willing to engage in work-related contact | 0.95 | 0.19 | .35 | <.001 |
| Hiring decision | 0.58 | 0.33 | .29 | .073 |

*Note. N* = 188.

# Reviewed Experiment Methods

## Instructions for Spontaneous Stereotypes

“What are the first 6 characteristics or traits that spontaneously come to mind when you think about the applicant? These answers will be completely anonymous. We are interested in your immediate, gut reaction. There are no right or wrong responses. Please try to use single words (and no more than two words, for example, an adjective + noun) for each of your answers.”

## Prototypical Words for Dimension Embeddings

The words we used to create dimension embeddings are depicted in Table S8.

| **Table S8**  *Most Prototypical Words from the Competence, Warmth, Health and Uniqueness Dimensions, Based on Nicolas et al. (2022), Table S2* | | | | | | | |
| --- | --- | --- | --- | --- | --- | --- | --- |
| Competence | |  | Warmth | |  |  |  |
| Ability | Assertiveness |  | Morality | Sociability |  | Health | Uniqueness |
| smart | hard-working |  | greedy | nice |  | unhealthy | different |
| intelligent | lazy |  | selfish | friendly |  | healthy | normal |
| educated | determined |  | bad | mean |  | sick | average |
| slow | confident |  | honest | fun |  | crazy | unique |
| *Note*. The dimensions *ability* and *assertiveness* are facets of competence, the dimensions *morality* and *sociability* are facets of warmth. The dimension *uniqueness* is called “deviance” in Nicolas et al. (2022) and “uniqueness” in Nicolas et al. (2024). | | | | | | | |

## Exclusion Criteria Based on Open-Response Data Quality

The quality assessment of open responses was based on the content of emails and spontaneous stereotypes. Conspicuous open responses were flagged by three coders generating the misgendering data and the first author. Guided by these assessments and the coders’ notes, the first author finally rated data quality. The rationale for exclusion from all analyses included duplicates (not within but between cases), nonsensical responses (e.g., “Fjkf”, “Cloudy”, or incoherent text), answers entirely unrelated to the task, and answers in different languages. In many cases, multiple criteria applied. One response explicitly refused participation using hostile, insulting, and highly vulgar language, and another contained a defensive and confrontational refusal framed as resistance to perceived coercion; both were excluded.

# Reviewed Experiment Analyses

## Perception of Applicant’s Sex/Gender & Transness

Considering participants’ responses to the applicants, we report the assumptions underlying them. Perceptions of applicants’ sex/gender, rather than their identity, revealed a consistent male bias. In the no-request condition, almost three thirds of participants assumed to rate a male applicant, and in the gender-neutral condition, this remained the most common belief (see Table S9). Participants were also nearly twice as likely to explicitly indicate the applicant was trans in the feminine-request than in the masculine-request condition, but both at a substantially higher rate than in the no-request condition (see Table S9).

| **Table S9**  *Reviewed Experiment: Assumptions about applicants* | | | | |
| --- | --- | --- | --- | --- |
|  | Condition | | | |
| Measure | Gender-neutral  (*n* = 336) | Feminine (*n* = 309) | Masculine  (*n* = 311) | [No request]  (*n* = 319) |
| Perceived gender identity |  |  |  |  |
| Neither F nor M or both | 61.61% (207) | 2.59% (8) | 2.57% (8) | 12.23% (39) |
| Feminine | 16.37% (55) | 89.64% (277) | 1.61% (5) | 16.93% (54) |
| Masculine | 22.02% (74) | 7.77% (24) | 95.82% (298) | 70.85% (266) |
| Perceived gender/sex |  |  |  |  |
| Neither F nor M or both | 35.42% (119) | 6.47% (20) | 2.25% (7) | 9.72% (31) |
| Female | 16.96% (57) | 50.49% (156) | 9.32% (29) | 17.87% (57) |
| Male | 47.62% (160) | 43.04% (133) | 88.42% (275) | 72.41% (231) |
| Perceived transness | 41.37% (139) | 40.78% (126) | 20.58% (64) | 7.21% (23) |

##

## Detailed View on Hiring Discrimination (Not Registered)

Beyond the forced choice format, coders categorized the decisions participants verbalized in the content of emails, because a substantial portion did not follow instructions to clearly verbalize rejection or hiring in their emails, but instead invited applicants to a job interview. Descriptive statistics are detailed in Table S10. Results of exploratory rank-based Kruskal-Wallis tests confirm those of the forced-choice format: The type of request influenced hiring decisions (response email: *H*(2, *N* = 903) = 15.95, *p* < .001, η^2^ = .015; email to colleague: *H*(2, *N* = 912) = 8.08, *p* =.018), η^2^ = .005). Bonferroni-corrected post-hoc tests (Dunn’s test) showed that applicants with a gender-neutral request were hired less often than masculine-request applicants (response: *z* = 3.42, *p_adj_* = .002, *r* = .14; colleague: *z* = 2.40,
*p_adj_* = .050, *r* = .10). Further, feminine-request applicants were hired less often than masculine-request applicants (response: *z* = 3.51, *p_adj_* = .001, *r* = .15; colleague: *z* = 2.54,
*p_adj_* = .034, *r* = .11).

| **Table S10**  *Reviewed Experiment: Descriptive statistics of hiring decision coded based on email content* | | | | |
| --- | --- | --- | --- | --- |
|  | Condition | | | |
| Measure | Gender-neutral | Feminine | Masculine | [No request] |
| Response email |  |  |  |  |
| Rejected: | 26.02% (83) | 24.83% (72) | 13.61% (40) | 12.33% (36) |
| Job interview: | 37.62% (120) | 40.00% (116) | 40.14% (118) | 43.15% (126) |
| Trial/almost hired: | 2.51% (8) | 3.79% (11) | 4.08% (12) | 4.11% (12) |
| Hired: | 33.86% (108)  *n* = 319 | 31.38% (91)  *n* = 290 | 42.18% (124)  *n* = 294 | 40.41% (118)  *n* = 292 |
| Email to colleague |  |  |  |  |
| Rejected: | 26.46% (86) | 25.09% (73) | 15.54% (46) | 12.62% (38) |
| Job interview: | 22.77% (74) | 24.40% (71) | 27.36% (81) | 26.58% (80) |
| Trial/almost hired: | 4.31% (14) | 6.87% (20) | 4.39% (13) | 7.64% (23) |
| Hired: | 46.46% (151)  *n* = 325 | 43.64% (127)  *n* = 291 | 52.70% (156)  *n* = 296 | 53.16% (160)  *n* = 311 |
| *Note*. Each email was coded into one category only. Unclear categorizations were counted as missing. | | | | |

Results for the response email held up to all robustness checks. Results for the email to the colleague, like the results for the forced-choice format, held up to all (including a memory check of the request) but those checks that excluded participants who misidentified the applicant’s gender identity.

In the participant subsample with experience in personnel selection, applicants with gender-neutral request were rejected more often but also hired more often than feminine-request applicants, whereas in the subsample without experience feminine-request applicants were rejected more often and hired less often than them. In both subsamples descriptive patterns for comparisons with the masculine-request condition follow H1, especially comparing rejection frequencies. However, post-hoc comparisons were only significant in the response email of participants without experience (*z* = 2.53, *p_adj_* = .034, *r* = .16). For response emails for participants with at least some experience the test just missed significance (*z* = 2.38, *p_adj_* = .051, *r* = .13).

Comparisons with the no-request condition show discrimination against applicants with a gender-neutral request (response email: *W* = 39882, *p* = .001, *r*_rb_ = .11; email to colleague: *W* = 42590, *p* =.002), *r*_rb_ = .12).

##

## Misgendering Intention

| **Table S11**  *Overview of Descriptives and Statistical Tests of the Reviewed Experiment for Intentional Misgendering.* | | | | | |
| --- | --- | --- | --- | --- | --- |
|  | Condition | | |  | |
| Misgendering sample | Gender-neutral | Feminine | Masculine |  | *p* |
| Any email | 34.62% (63)  (*n* = 182) | 51.79% (29)  (*n* = 56) | 33.33% (4)  (*n* = 12) |  | .068  (*N* = 250) |
| excluding  gendered words | 40.16% (51)  (*n* = 127) | 62.16% (23)  (*n* = 37) | 33.33% (4)  (*n* = 12) |  | .044  (*N* = 176) |
| Response email | 38.55% (32)  (*n* = 83) | 58.33% (21)  (*n* = 36) | 40.00% (2)  (*n* = 5) |  | .148  (*N* = 124) |
| excluding  gendered words | 55.88% (30)  (*n* = 68) | 66.67% (10)  (*n* = 27) | 40.00% (2)  (*n* = 5) |  | .134  (*N* = 100) |
| Email to colleague | 36.13% (56)  (*n* = 155) | 53.19% (25)  (*n* = 47) | 12.50% (1)  (*n* = 8) |  | .034  (*N* = 210) |
| excluding  gendered words | 40.66% (37)  (*n* = 91) | 56.67% (17)  (*n* = 30) | 12.50% (1)  (*n* = 8) |  | .061  (*N* = 129) |
| *Note*. Fisher’s exact tests for the three conditions with a request for specific use of pronouns/formal address (gender-neutral, feminine, masculine). Bonferroni-corrected post-hoc tests were not significant. Frequencies show how many participants, among those who misgendered, did not want to correct any mistakes to follow the applicant’s request. | | | | | |

## Politeness in Response Emails

Descriptives for H2c (word count/interaction length) and H2d (positive and negative affect) are depicted in Table 5.

*Word count* did not differ significantly between conditions (see Table 5), nor did it differ in the robustness checks or in the participant subgroup with at least some experience in personnel selection. Within this experienced subgroup, word count was descriptively highest in the feminine-request condition (*M* = 39.30, *SD* = 20.65), whereas the gender-neutral (*M* = 36.30, *SD* = 17.83) and masculine (*M* = 35.62, *SD* = 20.15) conditions showed similar word counts. However, in the no-experience subgroup, word count was significantly higher for applicants with a gender-neutral request (*M* = 40.78, *SD* = 28.68) compared to those with feminine (*M* = 34.10, *SD* = 15.93) or masculine (*M* = 33.66, *SD* = 14.98) request (*F*(2, 375) = 4.65, *p* = .010, η^2^ = .02), as Bonferroni-corrected post-hoc tests revealed (feminine: *p_adj_* = .040, *d* = .32; masculine: *p_adj_* = .004, *d* = .10). Therefore, H2c was not supported.

*Positive affect* shown in response emails (values obtained with LIWC) differed between conditions (see Table 5), but contrary to H2d expectations: Although affect shown to feminine-request applicants was the most positive, affect towards applicants with a gender-neutral request was on a similar level as for feminine-request applicants (*p_adj_* < .333) and *more* positive than for masculine-request applicants (*p_adj_* < .001, *d* = .28). This pattern did not change for robustness checks and the subgroup of participants with at least some experience in personnel selection. However, in the subgroup of participants without experience, the pattern changed: Gender-neutral-request applicants were met with similarly low levels of positive affect as masculine-request applicants (feminine: *p_adj_* = .040, *d* = .32; masculine:
*p_adj_* = 1), hinting at discriminatory tendencies. Therefore, H2d for positive affect was only partially supported in the no-experience subgroup and not at all in other subsamples.

*Negative affect* shown in response emails was descriptively highest in the gender-neutral condition (see Table 5) but absent in most emails (*Mdn* = 0 in all conditions, G-N: 76.49%, F: 75.73%, M: 85.85%). Kruskal-Wallis Test was significant (see Table 5). Bonferroni-corrected post-hoc tests indicate that masculine-request applicants were met with less negative affect than applicants with a gender-neutral request (*z* = -2.90, *p_adj_* = .004, *r* =
-.11), as expected. However, negative affect towards feminine-request applicants did not differ from that towards gender-neutral applicants (*z* = 0.13, *p_adj_* = 1, *r* = .01). Thus, H2d for negative affect was only partially supported. In all robustness checks, except those excluding participants who misidentified applicant gender identity, the patterns remained the same. The patterns also held in the participant subgroup with experience, although the difference between the gender-neutral vs. masculine condition was not significant (*z* = -2.29, *p_adj_* = .067, *r* = -.12). In the robustness checks excluding participants who misidentified applicant gender identity, as well as in the no-experience subgroup, the difference between the gender-neutral- vs. masculine-request condition was not significant (RC: *z* = -1.94, *p_adj_* = .156, *r* = -.09; no experience: *z* = -1.84, *p_adj_* = .197, *r* = -.11) and feminine-request applicants were met with the most negative affect.

## Covariates

### Main Effects Across Conditions

Including covariates did not change the pattern of results. For main effects, see Tables S12-14.

| **Table S12**  *Reviewed Experiment: Main Effects of Covariates on Formal Hiring Discrimination Across Three Conditions (Additive Model)* | | | | |
| --- | --- | --- | --- | --- |
|  | *B* | *SE* | β | *p* |
| *Hiring* | |  |  |  |
| Intercept  (ref: gender-neutral request) | 0.93 | 0.15 | .93 |  |
| Dummy feminine request | 0.08 | 0.19 | – | .685 |
| Dummy masculine request | 0.64 | 0.20 | – | .001 |
| ONBG | 0.30 | 0.05 | .54 | <.001 |
| Motivation to act non-prejudiced | 0.80 | 0.15 | .49 | <.001 |
| Political orientation (right) | -0.02 | 0.04 | -.04 | .664 |
| Participant gender (binary) (dummy: men) | -0.24 | 0.16 | – | .138 |
| *Hiring: experience subgroup* | | | | |
| Intercept | 0.95 | 0.20 | .95 |  |
| Dummy feminine request | -0.25 | 0.24 | – | .296 |
| Dummy masculine request | 0.49 | 0.26 | – | .056 |
| ONBG | 0.33 | 0.07 | .60 | <.001 |
| Motivation to act non-prejudiced | 1.07 | 0.21 | .67 | <.001 |
| Political orientation (right) | 0.05 | 0.06 | .10 | .418 |
| Participant gender (men) | -0.09 | 0.21 | – | .674 |
| *Hiring: no experience subgroup* | | | | |
| Intercept | 1.06 | 0.24 | 1.06 |  |
| Dummy feminine request | 0.19 | 0.30 | – | .530 |
| Dummy masculine request | 0.68 | 0.32 | – | .036 |
| ONBG | 0.23 | 0.08 | .43 | .112 |
| Motivation to act non-prejudiced | 0.37 | 0.23 | .23 | .083 |
| Political orientation (right) | -0.13 | 0.07 | -.26 | .076 |
| Participant gender (men) | -0.46 | 0.26 | – | .006 |
| *Note.* ONBG: Openness towards nonbinary gender.  Experience subgroup includes participants with at least some experience in personnel selection.  Results are expressed in log-odds. Continuous predictors were mean-centered. | | | | |

| **Table S13**  *Reviewed Experiment: Main Effects of Covariates on Interpersonal Discrimination Across Three Conditions (Additive Model)* | | | | |
| --- | --- | --- | --- | --- |
|  | *B* | *SE* | β | *p* |
| *Misgendering in any email* | | | | |
| Intercept  (ref: gender-neutral request) | 0.10 | 0.14 | .10 |  |
| Dummy feminine request | -1.85 | 0.20 | – | <.001 |
| Dummy masculine request | -3.58 | 0.32 | – | <.001 |
| ONBG | -0.03 | 0.06 | -.05 | .666 |
| Motivation to act non-prejudiced | -0.66 | 0.17 | -.41 | <.001 |
| Political orientation (right) | 0.10 | 0.05 | .22 | .035 |
| Participant gender (binary) (dummy: men) | 0.22 | 0.18 | – | .220 |
| *Misgendering excluding gendered words* | | | | |
| Intercept | -0.56 | 0.15 | -.56 |  |
| Dummy feminine request | -1.58 | 0.22 | – | <.001 |
| Dummy masculine request | -2.78 | 0.32 | – | <.001 |
| ONBG | -0.06 | 0.07 | -.11 | .373 |
| Motivation to act non-prejudiced | -0.37 | 0.17 | -.23 | .033 |
| Political orientation (right) | 0.08 | 0.05 | .17 | .126 |
| Participant gender (men) | 0.10 | 0.19 | – | .580 |
| *Willingness to engage in work-related contact* | | | | |
| Intercept | 4.82 | 0.10 | -.03 |  |
| Dummy feminine request | 0.01 | 0.12 | *B =* .01 | .925 |
| Dummy masculine request | 0.12 | 0.12 | *B =* .07 | .305 |
| ONBG | 0.22 | 0.04 | .24 | <.001 |
| Motivation to act non-prejudiced | 0.84 | 0.10 | .30 | <.001 |
| Political orientation (right) | -0.04 | 0.03 | -.05 | .141 |
| Participant gender (men) | 0.01 | 0.10 | *B =*.01 | .904 |
| *Politeness: Word Count (Interaction Length)* | | | | |
| Intercept | 37.83 | 1.23 | .05 |  |
| Dummy feminine request | -0.14 | 1.53 | *B* = -.01 | .928 |
| Dummy masculine request | -2.80 | 1.49 | *B* = .16 | .062 |
| ONBG | -0.52 | 0.47 | .13 | .272 |
| Motivation to act non-prejudiced | 4.04 | 1.12 | .30 | <.001 |
| Political orientation (right) | 0.06 | 0.38 | -.01 | .883 |
| Participant gender (men) | -0.54 | 1.24 | *B* = -.20 | .662 |
| *Politeness: Positive Affect* | | | | |
| Intercept | 11.83 | 0.32 | .10 |  |
| Dummy feminine request | 0.55 | 0.38 | *B* = .11 | .151 |
| Dummy masculine request | -1.35 | 0.39 | *B* = -.28 | <.001 |
| ONBG | 0.24 | 0.11 | .09 | .032 |
| Motivation to act non-prejudiced | 0.38 | 0.32 | .05 | .241 |
| Political orientation (right) | 0.06 | 0.09 | .03 | .511 |
| Participant gender (men) | -0.48 | 0.32 | *B* = -.10 | .137 |
| *Politeness: Negative Affect* | | | | |
| Intercept | 0.79 | 0.12 | .02 |  |
| Dummy feminine request | -0.07 | 0.14 | *B* = -.04 | .600 |
| Dummy masculine request | -0.36 | 0.14 | *B* = -.20 | .009 |
| ONBG | -0.15 | 0.05 | -.15 | .002 |
| Motivation to act non-prejudiced | -0.55 | 0.14 | -.19 | <.001 |
| Political orientation (right) | -0.03 | 0.03 | -.04 | .372 |
| Participant gender (men) | 0.19 | 0.11 | *B* = .10 | .080 |
| *Note.* ONBG: Openness towards nonbinary gender. Politeness measures show results for the response emails to applicants. Affect describes the affect displayed in language, values obtained with LIWC. Continuous predictors were mean-centered. Linear regressions used robust standard errors (HC3). Results for Misgendering are expressed in log-odds. | | | | |

| **Table S14**  *Reviewed Experiment: Main Effects of Covariates on Perception Variables Across Three Conditions (Additive Model)* | | | | |
| --- | --- | --- | --- | --- |
|  | *B* | *SE* | β | *p* |
| *Competence* | | | | |
| Intercept  (ref: gender-neutral request) | 4.23 | 0.05 | .06 |  |
| Dummy feminine request | -0.02 | 0.06 | *B* = -.02 | .801 |
| Dummy masculine request | 0.04 | 0.06 | *B* = .05 | .476 |
| ONBG | 0.04 | 0.02 | .09 | .019 |
| Motivation to act non-prejudiced | 0.43 | 0.05 | .32 | < .001 |
| Political orientation (right) | 0.03 | 0.01 | .07 | .067 |
| Participant gender (binary) (dummy: men) | -0.12 | 0.05 | *B* = -.14 | .023 |
| *Warmth* | | | | |
| Intercept | 3.96 | 0.05 | -.01 |  |
| Dummy feminine request | 0.08 | 0.06 | *B* = .10 | .168 |
| Dummy masculine request | 0.09 | 0.06 | *B* = .11 | .150 |
| ONBG | 0.05 | 0.02 | .12 | .005 |
| Motivation to act non-prejudiced | 0.46 | 0.05 | .33 | < .001 |
| Political orientation (right) | -0.00 | 0.01 | -.01 | .886 |
| Participant gender (men) | -0.09 | 0.05 | *B* = -.10 | .092 |
| *Perceived Fit* | | | | |
| Intercept | 5.60 | 0.08 | .06 |  |
| Dummy feminine request | -0.01 | 0.10 | *B* = -.02 | .930 |
| Dummy masculine request | 0.22 | 0.10 | *B* = .05 | .026 |
| ONBG | 0.10 | 0.03 | .09 | < .001 |
| Motivation to act non-prejudiced | 0.69 | 0.09 | .32 | < .001 |
| Political orientation (right) | -0.00 | 0.02 | .07 | .898 |
| Participant gender (men) | -0.28 | 0.09 | *B* = -.14 | .001 |
| *Note.* ONBG: Openness towards nonbinary gender.  Continuous predictors were mean-centered and regressions used robust standard errors (HC3). | | | | |

### Exploratory Moderation Analyses (Not Registered)

Exploring interactions, we found moderating effects of political orientation (on the willingness to engage in work-related contact, perceived job fit and misgendering in the email to a colleague) and motivation to act non-prejudiced (on the willingness to engage in work-related contact, competence and warmth), see Tables S15-16 and Figures S2-5. Further, participant gender moderated the effect on hiring (see Figure S6).

Regarding *willingness to engage in work-related contact*, we found that participants with lower motivation to act non-prejudiced (- 1 *SD*) were more willing to engage in contact with applicants with a masculine than with a gender-neutral request (*b* = 0.49, *SE* = 0.18, *p_adj_* = .007). And participants with a more right-leaning political orientation (+ 1 *SD*) were more willing to engage in work-related contact with masculine vs. gender-neutral-request applicants (*b* = 0.45, *SE* = 0.18, *p_adj_* = .013).

| **Table S15**  *Reviewed Experiment: Explorative Analyses for Motivation to Act Non-Prejudiced, Political Orientation and (Binary) Participant Gender as Potential Moderators for Willingness to Engage in Work-Related Contact (Unregistered)* | | | | |
| --- | --- | --- | --- | --- |
|  | *B* | *SE* | β | *p* |
| *Motivation to act non-prejudiced (MNP):* | |  |  |  |
| Intercept | 4.81 | 0.09 | -0.03 | <.001 |
| Gender-neutral – Feminine | 0.02 | 0.12 | 0.01 | .879 |
| Gender-neutral – Masculine | 0.12 | 0.12 | 0.07 | .292 |
| MNP (moderator) | 1.10 | 0.14 | 0.40 | <.001 |
| Political orientation (covariate) | -0.04 | 0.03 | -0.05 | .098 |
| Gender (Men – Women) (covariate) | 0.01 | 0.10 | 0.01 | .916 |
| ONBG (covariate) | 0.22 | 0.04 | 0.24 | <.001 |
| Gender-neutral – Feminine:MNP | -0.27 | 0.19 | -0.10 | .154 |
| Gender-neutral – Masculine:MNP | -0.60 | 0.20 | -0.22 | .003 |
| *Political Orientation:* | | | |  |
| Intercept | 4.81 | 0.10 | -0.03 | <.001 |
| Gender-neutral – Feminine | 0.01 | 0.12 | 0.01 | .901 |
| Gender-neutral – Masculine | 0.13 | 0.12 | 0.08 | .276 |
| Political Orientation (moderator) | -0.10 | 0.04 | -0.13 | .018 |
| MNP (covariate) | 0.85 | 0.10 | 0.31 | <.001 |
| Gender (Men – Women) (covariate) | 0.02 | 0.10 | 0.01 | .842 |
| ONBG (covariate) | 0.23 | 0.04 | 0.24 | <.001 |
| Gender-neutral – Feminine:Political Or. | 0.05 | 0.06 | 0.06 | .412 |
| Gender-neutral – Masculine:Political Or. | 0.15 | 0.06 | 0.19 | .007 |
| *(Binary) Participant Gender:* |  |  |  |  |
| Intercept | 4.82 | 0.11 | -0.02 | <.001 |
| Gender-neutral – Feminine | 0.04 | 0.16 | 0.02 | .806 |
| Gender-neutral – Masculine | 0.09 | 0.16 | 0.05 | .595 |
| Gender (Men – Women) (moderator) | 0.01 | 0.17 | 0.01 | .957 |
| MNP (covariate) | 0.84 | 0.10 | 0.30 | <.001 |
| Political orientation (covariate) | -0.04 | 0.03 | 0.24 | .144 |
| ONBG (covariate) | 0.22 | 0.04 | 0.24 | <.001 |
| Gender-neutral – Feminine: Gender | -0.06 | 0.24 | -0.04 | .794 |
| Gender-neutral – Masculine: Gender | 0.07 | 0.24 | 0.04 | .772 |
| *Note.* Analyses comparing the three main conditions. Continuous predictors were mean-centered and regressions used robust standard errors (HC3). | | | | |


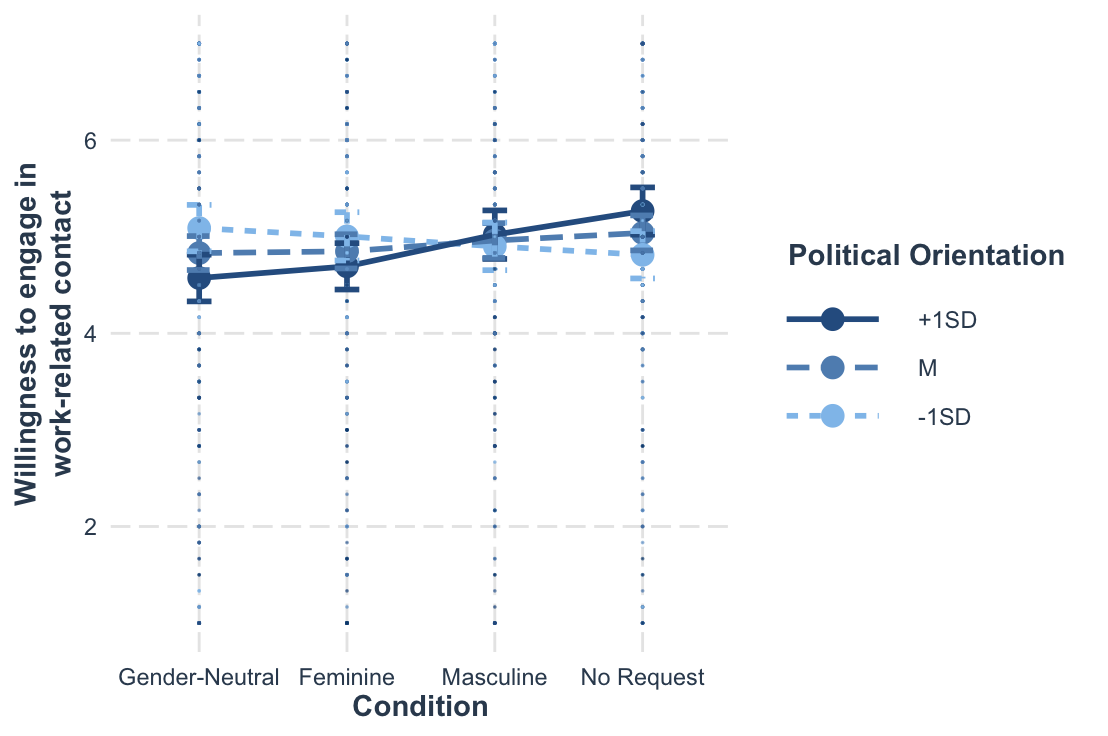

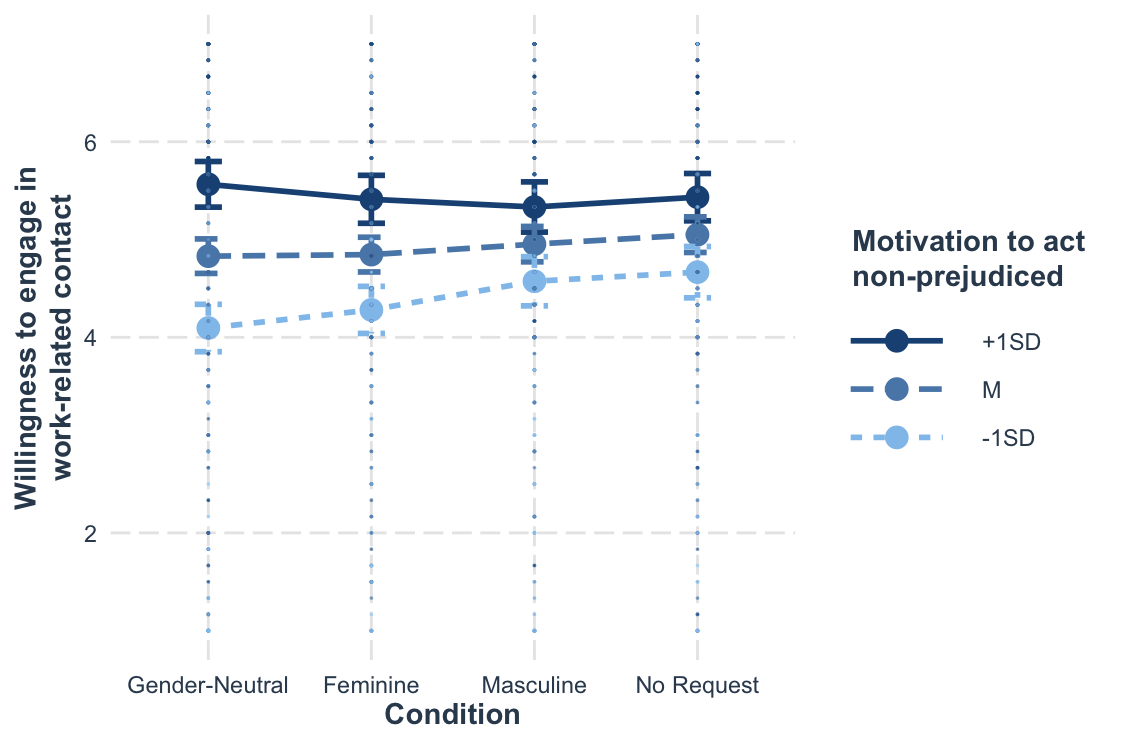
*Note.* Depicted are predicted values of the willingness to engage in contact.

**Figure S2**

*Reviewed Experiment: Estimated Marginal Means for the Moderating Effect of the Motivation to Act Non-Prejudiced and Political Orientation Regarding the Willingness to Engage in Work-Related Contact in All Conditions.*

Regarding *stereotype content*, we found that participants with lower motivation to act non-prejudiced (- 1 *SD*) rated masculine-request applicants higher in competence (*b* = 0.22, *SE* = 0.11, *p_adj_* = .037) and in warmth (*b* = 0.24, *SE* = 0.10, *p_adj_* = .018) than those with a gender-neutral request (see Table S16; Figure S3).

| **Table S16**  *Reviewed Experiment: Exploratory Moderation Analyses for Motivation to Act Non-Prejudiced (MNP) on Competence and Warmth (Unregistered)* | | | | | | | | | |
| --- | --- | --- | --- | --- | --- | --- | --- | --- | --- |
| Predictor | Competence | | | |  | Warmth | | | |
|  | *B* | *SE* | β | *p* |  | *B* | *SE* | β | *p* |
| Intercept | 4.23 | 0.05 | 0.06 | <.001 |  | 3.96 | 0.05 | -0.02 | <.001 |
| Gender-neutral – Feminine | -0.01 | 0.06 | -0.01 | .839 |  | 0.09 | 0.06 | 0.10 | 0.15 |
| Gender-neutral – Masculine | 0.05 | 0.06 | 0.05 | .454 |  | 0.09 | 0.06 | 0.10 | 0.13 |
| MNP | 0.58 | 0.07 | 0.42 | <.001 |  | 0.58 | 0.07 | 0.42 | <.001 |
| Political orientation | 0.02 | 0.01 | 0.06 | .086 |  | -0.00 | 0.01 | -0.01 | .793 |
| Men – Women | -0.12 | 0.05 | -0.14 | .022 |  | -0.09 | 0.05 | -0.10 | .088 |
| ONBG | 0.04 | 0.02 | 0.09 | .018 |  | 0.05 | 0.02 | 0.12 | .002 |
| Gender-neutral – Feminine:MNP | -0.18 | 0.10 | -0.13 | .076 |  | -0.15 | 0.10 | -0.11 | .125 |
| Gender-neutral – Masculine:MNP | -0.29 | 0.10 | 0.21 | .004 |  | -0.25 | 0.10 | -0.18 | .015 |
| *Note.* Competence: *F*(8, 932) = 19.64, *p* < .001, *R*^2^ = .14.  Warmth: *F*(8, 932) = 26.05, *p* < .001, *R*^2^ = .18. Continuous predictors were mean-centered and regressions used robust standard errors (HC3). | | | | | | | | | |


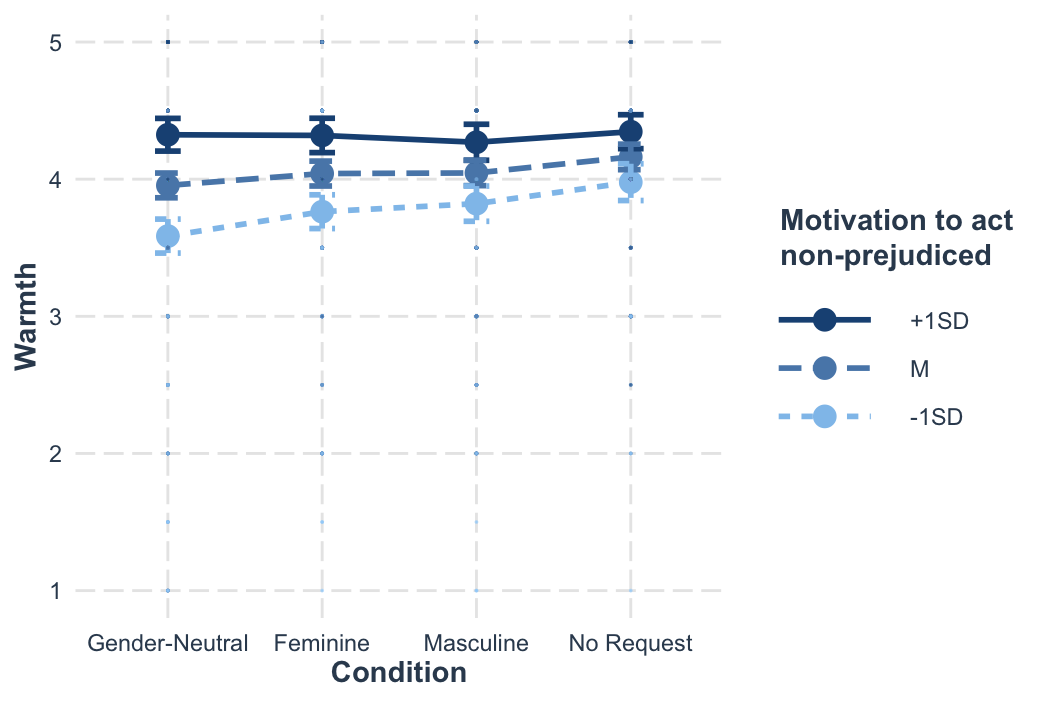

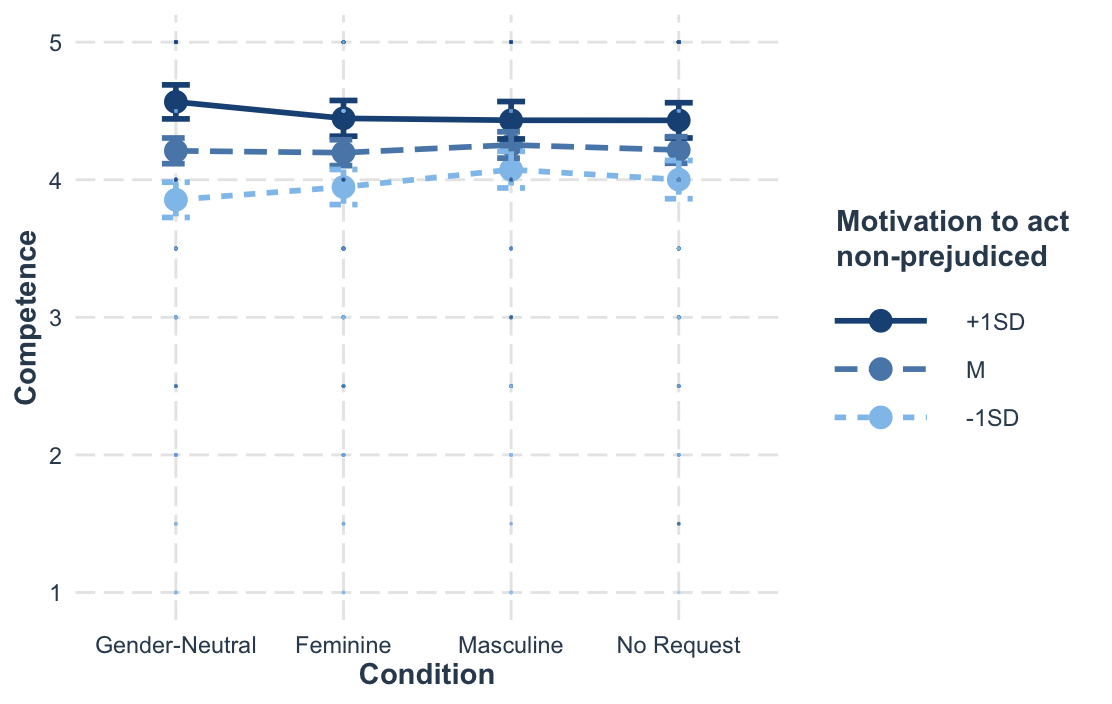


*Note.* Depicted are predicted values of competence and warmth.

**Figure S3**

*Reviewed Experiment: Estimated Marginal Means for the Moderating Effect of the Motivation to Act Non-Prejudiced Regarding Competence and Warmth in All Conditions*


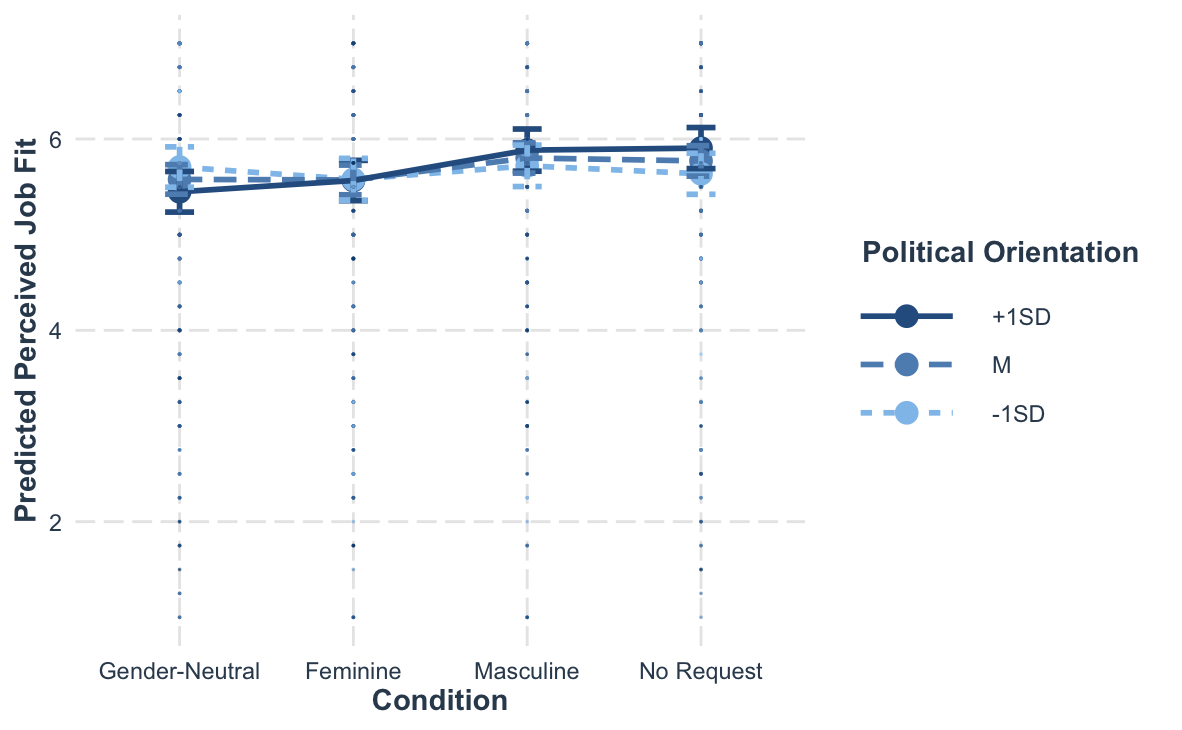
For *perceived job fit*, we found that participants with a more right-leaning political orientation (+ 1 *SD*) rated masculine-request applicants higher in perceived fit than those with a gender-neutral request (*b* = 0.44, *SE* = 0.15, *p_adj_* = .005; see Figure S4). The motivation to act non-prejudiced followed the pattern of H4 for ONBG, but the interaction for the gender-neutral vs. masculine-request comparison was not significant (*b* = -0.35, *p* = .057).

**Figure S4**

*Reviewed Experiment: Estimated Marginal Means for the Moderating Effect of Political Orientation Regarding Perceived Fit*

Regarding *misgendering,* simple slopes analysis revealed that request type was a significant predictor of misgendering at all levels of political orientation. For more left-leaning participants (- 1 *SD*), both the feminine-request (*b* = -1.61, *p_adj_* < .001, *OR* = 0.20, excluding gendered words: *b* = -1.55, *p_adj_* < .001, *OR* = 0.21) and masculine-request (*b* = -2.60, *p_adj_* < .001, *OR* = 0.07, excluding gendered words: *b* = -2.00, *p_adj_* < .001, *OR* = 0.14) conditions significantly reduced the odds of misgendering relative to the neutral condition. For more right-leaning individuals (+1 *SD*), the feminine-request (*b* = -2.08,

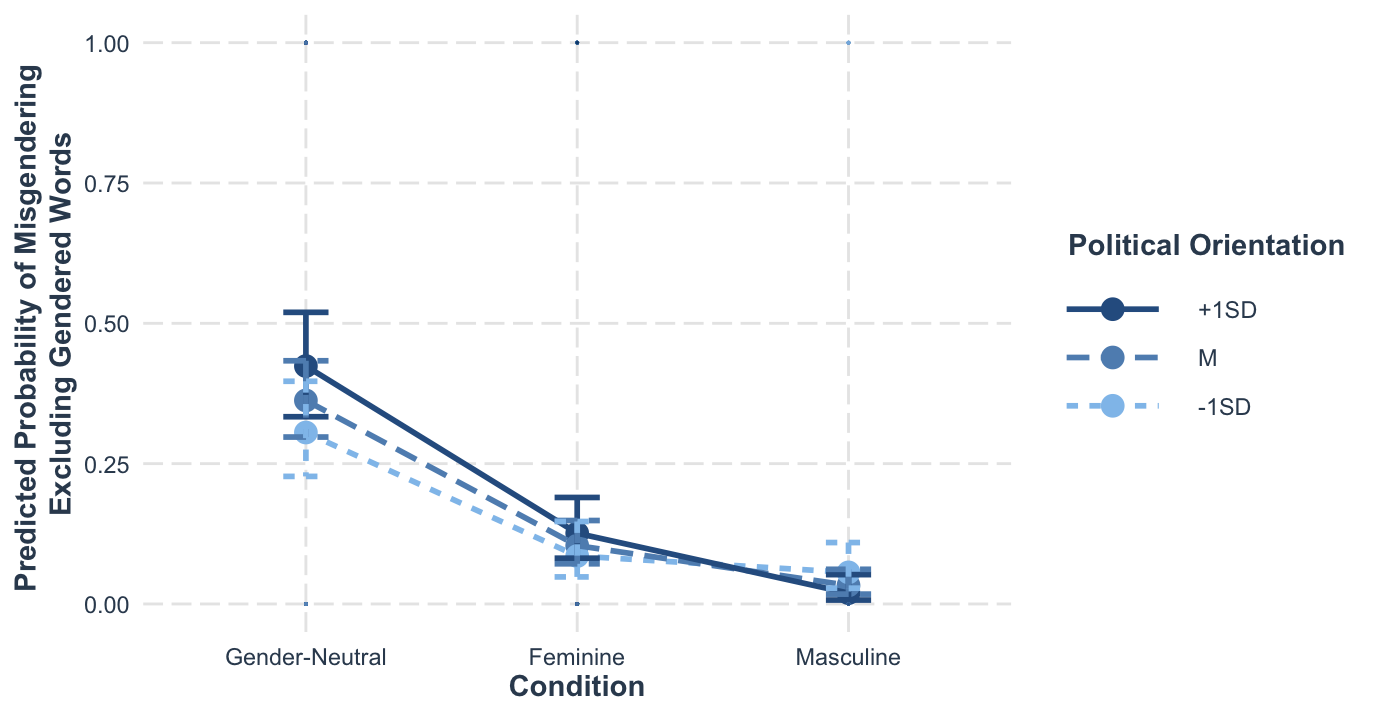

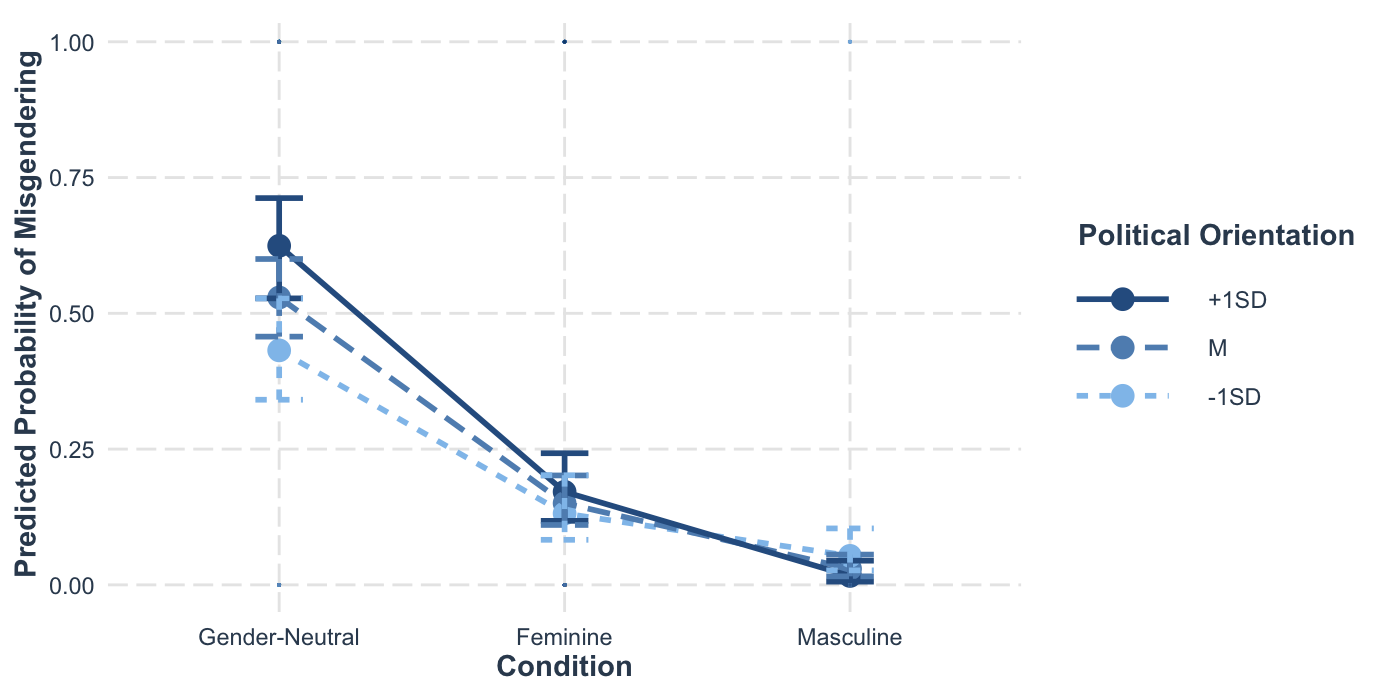
*p_adj_* < .001, *OR* = 0.12, excluding gendered words: *b* = -1.63, *p_adj_* < .001, *OR* = 0.20) and masculine-request (*b* = -4.60,  *p_adj_* < .001, *OR* = 0.01, excluding gendered words: *b* = -3.62,  *p_adj_* < .001, *OR* = 0.03) conditions continued to significantly reduce misgendering. Among moderate (*b* = -1.75, *p_adj_* < .001, *OR* = 0.17, excluding gendered words: *b* = -1.22, *p_adj_* = .003, *OR* = 0.30) and right-leaning individuals (*b* = -2.52,  *p_adj_* < .001, *OR* = 0.08, excluding gendered words: *b* = -1.99,  *p_adj_* = .001, *OR* = 0.14), misgendering odds for masculine-request applicants were also lower than those of feminine-request applicants. The strength of the masculine-request effect increased with political conservatism, indicating a moderating effect of political orientation (see Figure S5). Looking only at the email to the applicant, no interactions with political orientation were found (feminine-request: *b* = 0.08, *p* = .473, masculine-request: *b* = -0.03, *p* = .887).

**Figure S5**

*Reviewed Experiment: Estimated Marginal Means for the Moderating Effect of Political Orientation Regarding Misgendering*

*Hiring decision* for applicants with a feminine (vs. gender-neutral) request was moderated by participant gender (*b* = -0.80, *SE* = 0.37, *p* = .030) in surprising ways (see Figure 6): Among women, main results stayed the same, i.e., the gender-neutral condition was associated with significantly lower odds of selecting the applicant (*b* = -0.90, *SE* = 0.29, *p_adj_* = .005, *OR* = 0.41) relative to the masculine-request condition. Among men, feminine-request applicants had lower hiring odds than masculine-request applicants (*b* = -0.71, *SE* = 0.27, *p_adj_* = .028, *OR* = 0.49) and the difference between gender-neutral and masculine-request applicants was not significant (*b* = -0.39, *SE* = 0.27, *p_adj_* = .450, *OR* = 0.68).

**Figure S6**

*Estimated Marginal Means for the Moderating Effect of (Binary) Participant Gender Regarding Hiring*


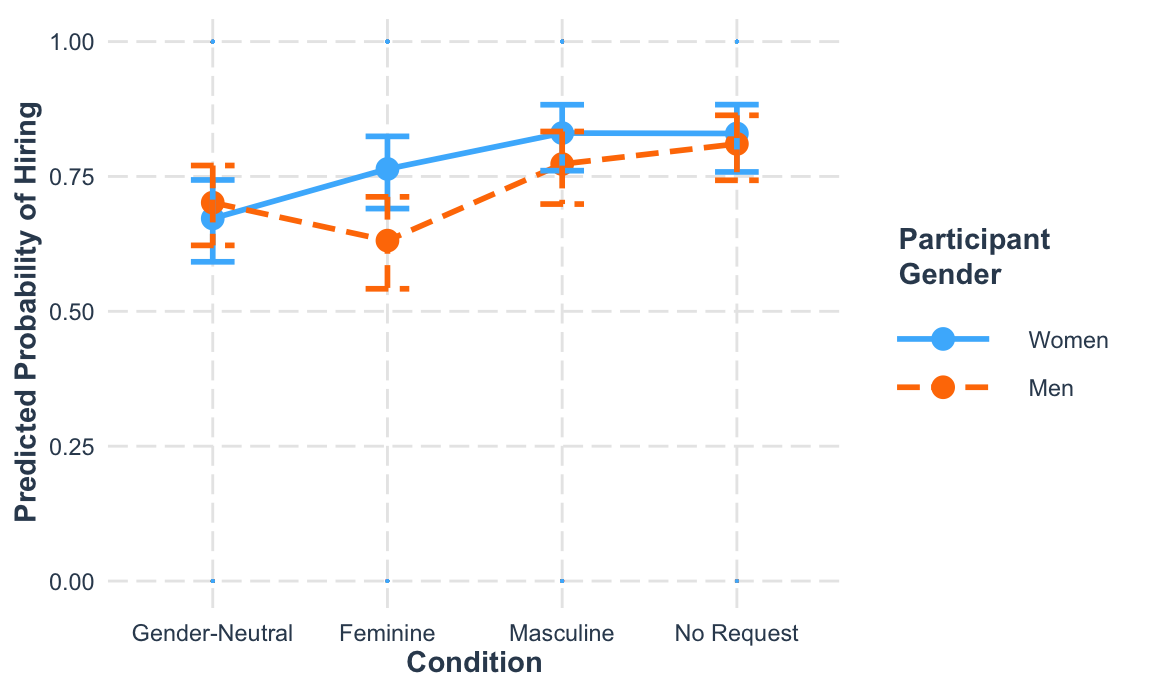


In sum, the motivation to act non-prejudiced led to similar result patterns as ONBG, political orientation was relevant for misgendering (and WWRC), and gender moderated hiring decisions.

## Openness Towards Nonbinary Gender (ONBG)

### Main Effects Across Conditions

Table S17 shows that ONBG influences all dependent variables except misgendering and word count across the gender-neutral, feminine-, and masculine-request conditions.

| **Table S17**  *Reviewed Experiment: Main Effects of Openness Towards Nonbinary Gender (ONBG) on Dependent Variables Across Three Conditions with all Covariates (Additive Model)* | | | | |
| --- | --- | --- | --- | --- |
|  | *B* | *SE* | β | *p* |
| Hiring | 0.30 | 0.05 | .54 | <.001 |
| Hiring: experience subgroup | 0.33 | 0.07 | .60 | <.001 |
| Hiring: no experience subgroup | 0.23 | 0.08 | .43 | .112 |
| Misgendering in any email | -0.03 | 0.06 | -.05 | .666 |
| excluding gendered words | -0.06 | 0.07 | -.11 | .373 |
| Willingness to engage in work-related contact | 0.22 | 0.04 | .24 | <.001 |
| Politeness: word count  (interaction length) | -0.52 | 0.47 | .13 | .272 |
| Politeness: positive affect | 0.24 | 0.11 | .09 | .032 |
| Politeness: negative affect | -0.15 | 0.05 | -.15 | .002 |
| Competence | 0.04 | 0.02 | .09 | .019 |
| Warmth | 0.05 | 0.02 | .12 | .005 |
| Perceived fit | 0.10 | 0.03 | .09 | < .001 |
| *Note.* Analyses included all covariates. Experience subgroup includes participants with at least some experience in personnel selection. Politeness measures show results for the response emails to applicants. Affect describes the affect displayed in language, values obtained with LIWC.  Continuous predictors were mean-centered. Linear regressions used robust standard errors (HC3). Results for hiring and misgendering are expressed in log-odds. | | | | |

### Moderation of ONBG on Competence, Warmth and Perceived Fit

Models testing the moderating role of openness toward nonbinary gender (ONBG) on the relationship between request type and perceived warmth/competence were statistically significant (warmth: *F*(5, 950) = 24.26, *p* < .001, *R*^2^ = 0.11; competence: *F*(5, 950) = 14.93,
*p* < .001, *R*^2^ = 0.07). For *warmth*, we found a significant interaction for the masculine vs. gender-neutral request condition (*b* = -0.13, *SE* = 0.04, *t*(950) = -3.06, *p* < .001). At higher ONBG (+1 *SD,* H4a) applicants with a masculine request were rated similarly in warmth (*b* = -0.18, *SE* = 0.09, *p_adj_* = .143) as in the gender-neutral request, and at lower ONBG levels (-1 *SD,* H4b) masculine-request applicants were rated higher in warmth (*b* = 0.31, *SE* = 0.09, *p_adj_* = .002) than gender-neutral-request applicants (see Figure 2d).

For *competence*, we found significant interactions with ONBG for applicants with a feminine (*b* = -0.09, *SE* = 0.04, *t*(950) = -2.40, *p* = .017) or masculine (*b* = -0.11, *SE* = 0.04, *t*(950) = -3.06, *p* = .002) vs. gender-neutral request. As ONBG decreased, the effect of request type (masculine vs. gender-neutral and less so feminine vs. gender-neutral) increased, but even at low ONBG (-1 *SD,* H4b) differences for the masculine-request condition were (marginally) not significant (*b* = 0.22, *SE* = 0.09, *p* = .054), indicating that competence was only higher for applicants with a masculine or feminine (vs. gender-neutral) request given very low ONBG.


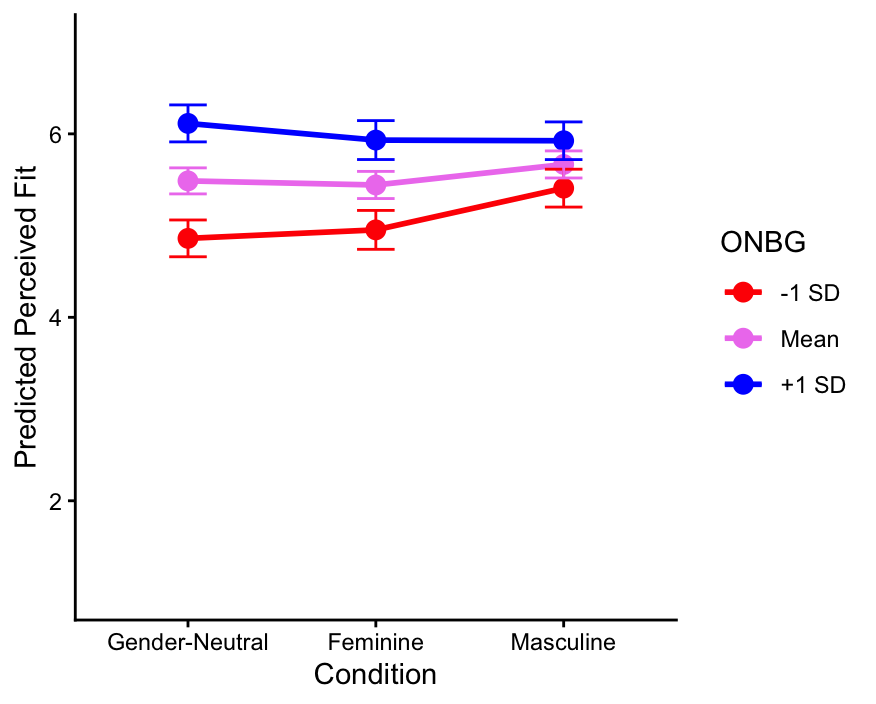
We examined the moderating role of ONBG on the effect on *perceived fit* (*F*(5, 950) = 14.93, *p* < .001, *R*^2^ = 0.12) and found an interaction effect for the masculine-request condition (*b* = -0.20, *SE* = 0.06, *t*(950) = -3.39, *p* < .001), see Figure S7. In line with the suggested pattern of H4, comparing the gender-neutral with the masculine-request condition, we found no significant differences for participants with higher ONBG (+1 SD; *b* = -0.19, *SE* = 0.15, *p_adj_* = .592), but a significant effect for participants with lower (-1 SD) levels of ONBG (*b* = 0.55, *SE* = 0.15, *p_adj_* < .001). Given low ONBG, feminine-request applicants were also perceived as less fitting than masculine-request applicants (*b* = 0.45, *SE* = 0.15, *p_adj_* = .008).

**Figure S7**

*Reviewed Experiment: Estimated Marginal Means for the Moderating Effect of the Openness Towards Nonbinary Gender (ONBG) Regarding Perceived Fit of Applicants*

## Mediation Analyses

Using PROCESS Model 14 (Hayes, 2022) with bootstrapping for mediation via perceived applicant warmth/competence, we tested whether an indirect effect of request type (gender-neutral vs. masculine) on perceived job fit or on hiring was moderated by the perceived warmth/competence required by the job position. We found effects of stereotype content on perceived fit directly (warmth: *b* = 1.19, *BootSE* = 0.05, 95% CI [1.10, 1.28]; competence: *b* = 1.20, *BootSE* = 0.04, 95% CI [1.11, 1.28]). The effect of applicant competence on perceived fit was moderated by job competence (*b* = 0.10, *BootSE* = 0.04, 95% CI [0.01, 0.18]), but we also found a direct effect of masculine (vs. gender-neutral) request on perceived fit *b* = 0.16, *BootSE* = 0.07, 95% CI [0.02, 0.30]). We further found effects of stereotype content on hiring (warmth: *b* = 2.15, *BootSE* = 0.17, 95% CI [1.88, 2.53], *OR* = 8.58; competence: *b* = 1.62, *BootSE* = 0.14, 95% CI [1.38, 1.93], *OR* = 5.05) but no interactions with job stereotype.

We then tested whether the effect of request type (masculine vs. gender-neutral) on hiring operated indirectly through perceived warmth/competence. Because ONBG was found to be a moderator of stereotype content, we deviated from our original path analyses based on Figure S1 and instead included ONBG as moderator (PROCESS Model 7).

The interaction between masculine (vs. gender-neutral) request and ONBG on *warmth* was significant (*b* = -0.10, *BootSE* = 0.03, 95% CI [-0.16, -0.04]), indicating that masculine request increased perceived warmth more at lower ONBG. Higher warmth strongly predicted greater odds of being hired (*b* = 2.04, 95% CI [1.77, 2.40], *OR* = 7.66), and masculine request also increased hiring odds directly (*b* = 0.57, 95% CI [0.12, 1.02], *OR* = 1.77). The conditional indirect effect at low ONBG (16^th^ percentile) was significant (Effect = 0.64, *BootSE* = 0.24, 95% CI [0.19, 1.11]), but not at moderate or high ONBG. The significant index of moderated mediation (Index = -0.21, *BootSE* = 0.07, 95% CI [-0.36, -0.08]) indicates that the indirect effect decreased as ONBG increased.

For *competence* as a mediator, the interaction between masculine (vs. gender-neutral) request type and ONBG significantly predicted competence (*b* = -0.07, *BootSE* = 0.03, 95% CI [-0.13, -0.00]) and perceived competence predicted greater hiring odds (*b* = 1.53,
*BootSE* = 0.13, 95% CI [1.31, 1.81], *OR* = 4.61). The direct effect of masculine request type on hiring remained significant (*b* = 0.61, *BootSE* = 0.21, 95% CI [0.20, 1.01], *OR* = 1.84), again suggesting partial mediation. The conditional indirect effect was small and even at low ONBG (16^th^ percentile) nonsignificant (Effect = 0.27, *BootSE* = 0.18, 95% CI [-0.07, 0.63]), but the index of moderated mediation was significant, albeit small (Index = -0.10, *BootSE* = 0.05, 95% CI [-0.21, -0.00]).


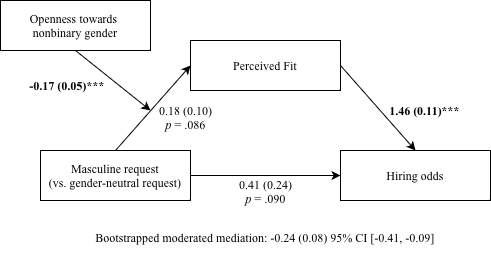
We tested whether the effect of request type (masculine vs. gender-neutral) on hiring operated indirectly through *perceived job fit* and again included ONBG as moderator (see “Moderation of ONBG on Competence, Warmth and Perceived Fit”). We found a full moderated mediation (see Figure S8). The interaction between request type and ONBG predicting perceived fit was significant (*b* = -0.17, *BootSE* = 0.05, 95% CI [-0.27, -0.06]). At lower levels of ONBG (16^th^ percentile), applicants with a masculine request were perceived as more fitting for the job (*b* = 0.58, *BootSE* = 0.16, 95% CI [0.27, 0.88]), but not at other levels. Perceived fit strongly predicted a higher likelihood of being hired (*b* = 1.46, *BootSE* = 0.11, 95% CI [1.28, 1.69], *OR* = 4.31), whereas the direct effect of request type was not significant (*b* = 0.41, *BootSE* = 0.24, 95% CI 0.94, 2.42], *OR* = 1.51). The indirect effect of request type on hiring via perceived fit was significant only at low ONBG, (Effect = 0.84, *BootSE* = 0.26, 95% CI [0.34, 1.37]). The index of moderated mediation was significant (Index = -0.24, *BootSE* = 0.08, 95% CI [-0.41, -0.09]), indicating that the indirect effect decreased as ONBG increased.

**Figure S8**

*Reviewed Experiment: Moderated Mediation on Hiring via Perceived Job Fit*

*Note.* Coefficients are unstandardized coefficients and bootstrapped standard errors. Continuous scales were mean-centered

## Spontaneous Stereotypes

### Text Embedding Analyses (H5)

We used FastText and in addition SBERT to obtain embeddings, with SBERT providing more accurate representations of multiword responses. For all FastText outcomes (and the SBERT outcomes for morality as a facet of warmth), the random intercept for the applicant’s name (i.e., stimulus) was removed due to singularity (variance ≈ 0). For other SBERT outcomes, random intercepts of both participant and applicant name were retained. See Table S18 for descriptives. Likelihood ratio tests comparing full to null models indicate that type of request significantly improved model fit for normality/uniqueness obtained with SBERT (χ²(2) = 7.76, *p* = .021) and only marginally for (un)health obtained with FastText (χ²(2) = 5.42, *p* = .066), warranting cautious interpretation. All other full models did not significantly differ from their respective null models.

Contrary to expectations (H5a), results for the linear mixed-effects model predicting *normality* showed higher similarities for both the feminine (*b* = 0.01, *SE* = 0.00, *t*(874) = 2.32, *p* = .020) and masculine-request conditions (*b* = 0.01, *SE* = 0.00, *t*(884) = 2.49, *p* = .013) compared to the gender-neutral request condition.

In line with expectations (H5b), results for the *health* (FastText) model indicate that embedding similarities were slightly lower in the feminine- than gender-neutral-request condition (*b* = -0.01, *SE* = 0.00, *t*(908) = -2.33, *p* = .020) whereas, not in line with H5b predictions, similarities did not significantly differ between the masculine- and gender-neutral-request condition (*b* = -0.00, *SE* = 0.00, *t*(914) = -1.10, *p* = .272). As noted above, these results should be interpreted cautiously.

In (registered) analyses exploring embedding similarities for competence and warmth, only SBERT embeddings for ability (competence facet), assertiveness (competence facet) and sociability (warmth facet) significantly differed from their respective null model. For *ability,* similarities were higher for both feminine-request (*b* = 0.01, *SE* = 0.01, *t*(881) = 2.19, *p* = .029) and masculine-request applicants (*b* = 0.02, *SE* = 0.01, *t*(890) = 3.91, *p* < .001) compared with gender-neutral-request applicants. For *assertiveness*, we found higher similarities for masculine-request applicants (*b* = 0.02, *SE* = 0.01, *t*(889) = 2.89, *p* = .004) than for gender-neutral-request applicants, whereas the comparison with feminine-request applicants was not significant (*b* = 0.01, *SE* = 0.01, *t*(880) = 2.19, *p* = .056). For *sociability*, the pattern was similar to ability (feminine: *b* = 0.01, *SE* = 0.01, *t*(890) = 2.56, *p* = .011, masculine: *b* = 0.02, *SE* = 0.01, *t*(899) = 2.75, *p* = .006). This indicates that, in the condition with a gender-neutral request, words describing applicants were less similar to ability, (partially) assertiveness and sociability suggesting that descriptions related to other dimensions or unique stereotypes more.

| **Table S18**  *Means and Standard Deviations of the Reviewed Experiment for Spontaneous Stereotypes (Embedding Similarities; H5)* | | | | | | | | |
| --- | --- | --- | --- | --- | --- | --- | --- | --- |
|  | Condition | | | | | | | |
| Dimension | Gender-neutral  (*n* = 336) | | Feminine (*n* = 309) | | Masculine  (*n* = 311) | | [No request]  (*n* = 319) | |
|  | *M* | *SD* | *M* | *SD* | *M* | *SD* | *M* | *SD* |
| a) *Normality* |  |  |  |  |  |  |  |  |
| FastText | .37 | .12 | .37 | .11 | .37 | .11 | .35 | .11 |
| SBERT | .53 | .12 | .54 | .11 | .54 | .11 | .54 | .11 |
| b) *Health* |  |  |  |  |  |  |  |  |
| FastText | .35 | .10 | .34 | .09 | .35 | .09 | .33 | .09 |
| SBERT | .47 | .12 | .48 | .12 | .47 | .11 | .47 | .12 |
| *Competence* | | | | | | | | |
| Ability |  |  |  |  |  |  |  |  |
| FastText | .42 | .13 | .42 | .13 | .43 | .13 | .41 | .13 |
| SBERT | .62 | .14 | .63 | .13 | .64 | .13 | .64 | .13 |
| Assertiveness |  |  |  |  |  |  |  |  |
| FastText | .39 | .12 | .38 | .12 | .39 | .11 | .38 | .11 |
| SBERT | .64 | .13 | .65 | .13 | .65 | .12 | .65 | .12 |
| *Warmth* | | | | | | | | |
| Morality |  |  |  |  |  |  |  |  |
| FastText | .42 | .13 | .41 | .13 | .42 | .12 | .40 | .13 |
| SBERT | .55 | .12 | .56 | .12 | .56 | .12 | .56 | .12 |
| Sociability |  |  |  |  |  |  |  |  |
| FastText | .44 | .16 | .43 | .16 | .44 | .16 | .43 | .17 |
| SBERT | .60 | .14 | .61 | .14 | .61 | .14 | .61 | .14 |
| *Note*. FastText indicates the use of word embeddings from the FastText model. SBERT indicates the use of text embeddings from the SBERT model.  Values are cosine similarities between dimension and text embeddings. | | | | | | | | |

### Raw Data Patterns of Spontaneous Stereotypes

Inspection of raw data suggests that embedding results might not accurately represent the data: Descriptively, the number of words describing normality/uniqueness as well as (un)health show a distinct pattern in line with H5, with the highest number in the gender-neutral-request condition, followed by the feminine-request condition and lowest for the masculine-request condition. The no request condition was similarly low in normality/ uniqueness words as the masculine-request condition, but words describing (un)health were completely absent.

## Content of Emails (Not Registered)

In addition to testing the preregistered hypotheses, we explored the content of the e-mails written to get insight into reasons of rejection. Participants who mentioned the experimental manipulation, either directly (“the P.S.”) or indirectly through pronouns, gender identity, or communication, and those who referred to personality or other relevant topics (e.g., job fit) in (mostly) emails to the substitute, were most often found in the gender-neutral-request condition, followed by the feminine-request condition. While some explicitly called the applicant mentally ill or complicated and some more indirectly as “not fitting”, a recurring justification was that, despite being qualified, the applicant was rejected because they were seen as potentially problematic for customer interactions and team cohesion. Another noteworthy theme concerned a divide among participants: Some saw the request as a sign that the applicant was overly focused on their gender identity, whereas others demanded official gender proof or insisted that the applicant should “decide on” a gender. Positive references to the manipulation were comparably fewer and mostly consisted of a reminder to the colleague to use the correct language in communication, sometimes also in official forms.
